# Tissue‐specific gene delivery approaches

**DOI:** 10.1002/btm2.70125

**Published:** 2026-02-24

**Authors:** Sarah S. Nasr, Yahya Cheema, Alexa Stern, Owen Tabah, Stephanie Poore, Gregg A. Duncan

**Affiliations:** ^1^ Fischell Department of Bioengineering University of Maryland College Park Maryland USA; ^2^ Department of Pharmaceutics Alexandria University Alexandria Egypt

**Keywords:** adeno‐associated virus, gene delivery, nanoparticles, organ targeting, protein corona

## Abstract

For genetic therapies to have their intended benefit, delivery systems must be designed which reach disease‐affected organs with high efficiency. To accomplish this, gene delivery systems must overcome multiple intra‐ and extracellular barriers to avoid rapid clearance from the body and/or significant accumulation in off‐target sites which can lead to undesired side effects (e.g., genotoxicity, immunogenicity). This requires an in‐depth knowledge of biomolecular and biophysical interactions at the nano–bio interface to engineer gene vectors which preferentially access specific organs such as the liver, spleen, and brain after systemic administration. In this review, we will discuss the strategies employed to engineer genetic therapies which selectively target organs of interest after systemic administration. We focus on three major classes of nucleic acid delivery systems including adeno‐associated viruses, lipid nanoparticles, and polymeric nanoparticles (PNPs) which are all being explored for tissue‐specific gene delivery. We will go on to describe how new, highly efficient adeno‐associated virus variants as well as engineered lipid and PNPs can be discovered or rationally designed. We also discuss high throughput approaches for screening of these systems to establish important structure‐to‐function relationships that determine the fate of these gene delivery systems once administered.

## Graphical abstract

This review synthesizes emerging principles for engineering organ‐specific gene delivery across viral and non‐viral platforms. By integrating nano–bio interactions, physiological barriers, and targeting mechanisms, it highlights strategies that improve tissue selectivity, therapeutic potency, and safety, guiding the rational design of next‐generation genetic therapies.

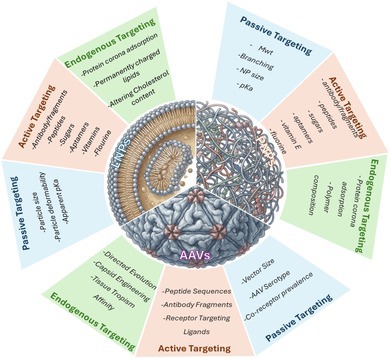

**Translational Impact Statements**

The ability to safely and efficiently deliver genetic therapies to specific organs is a critical bottleneck in bringing next‐generation treatments to patients. This review covers the leading gene delivery systems—including adeno‐associated viruses, lipid nanoparticles, and polymeric nanoparticles—used to overcome biological barriers and achieve precise tissue targeting after systemic administration. By highlighting approaches for rational vector design, high‐throughput screening, and structure–function optimization, this work provides a roadmap for translating molecular insights at the nano–bio interface into clinically relevant gene delivery platforms.

## 1 INTRODUCTION

The landscape of newly approved drug products has dramatically shifted over the past two decades with the emergence of cell and genetic therapies.^1^ The onset of the coronovirus disease 2019 (COVID‐19) pandemic followed by the subsequent deployment of mRNA‐based vaccines^2^ further triggered a surge in preclinical and clinical research investigating gene therapies for protein replacement, genome editing, and in vivo generated cell‐based therapies.^3^ Since nucleic acids (NAs) are hydrophilic and negatively charged macromolecules, they cannot cross the cell membrane efficiently in a soluble (naked) form. As such, their internalization in target cells heavily relies on nanocarriers for successful delivery. Packaging of nucleic acid cargos is most typically accomplished using (i) viruses that are innately able to deliver genes into mammalian cells or (ii) nanoparticle (NP) systems commonly composed of lipids, natural polymers, or synthetic polymers. Nanocarriers dictate the safety, efficiency, and targeting of nucleic acid therapies.^4^ To realize the significant potential of in vivo nucleic acid therapeutics, we must engineer next‐generation nanocarriers to effectively deliver nucleic acids to target sites.

To summarize the status in clinical development of organ‐targeted gene delivery systems, we have generated a list of gene therapies based upon the following criteria: encapsulate nucleic acids in relevant nanocarriers, are administered systemically by intravenous injection, target specific organs or tissues, and have Food & Drug Administration (FDA) approval or are in ongoing clinical trials (Table 1). The first‐ever FDA approved in vivo gene therapy was released in 2017 using adeno‐associated virus (AAV) as a gene delivery platform administered intraocularly.^24^, ^25^ Several organ‐targeted AAV gene therapy products have gained clinical approval to treat conditions such as Duchenne muscular dystrophy (DMD), hemophilia A/B, and spinal muscular atrophy.^26^ At least 12 known naturally occurring AAV serotypes have been identified with a broad range of tissue tropisms. As a result, AAV gene therapies are widely tested and under development for tissue‐specific gene delivery to organs including the brain, liver, and lung.^27^, ^28^ However, systemic delivery of AAV has been met with significant challenges due to toxicity concerns when delivered at the high doses required to achieve therapeutic efficacy. To minimize off‐target side effects, significant strides have been made in engineering AAVs with enhanced tropism to specific organs and to further reduce their immunogenicity to enable repeated dosing.

**TABLE 1:** Summative list of clinical phase organ‐targeted, intravenously injected nanocarrier enabled gene therapies. Definitions for abbreviations used—*Diseases*: Duchenne muscular dystrophy = DMD; heterozygous familial hypercholesterolemia = HeFH; PCAD = premature coronary artery disease. *Vectors*: AAV = adeno‐associated virus; LNP = lipid nanoparticle; LV = lentivirus. Cargo: single stranded DNA = ssDNA; single guide RNA = sgRNA; Clustered Regularly Interspaced Short Palindromic Repeats (CRISPR)‐associated protein 9 = Cas9. *Targeted Tissues*: central nervous system (CNS). *Targeting modes*: P = passive; E = endogenous; A = active.

Trade name	Disease	Vector/cargo	Targeted tissue(s)	Targeting mode(s)	Clinical phase	References
Onasemnogene abeparvovec (Zolgensma)	Spinal muscular atrophy	AAV9/ssDNA	CNS	P, E	FDA approved	5
Etranacogene dezaparvovec (Hemgenix)	Hemophilia B	AAV5/ssDNA	Liver	P, E	FDA approved	5
Valoctocogene roxaparvovec (Roctavian)	Hemophilia A	AAV5/ssDNA	Liver	P, E	FDA approved	5
Delandistrogene moxeparvovec (Elevidys)	DMD	AAVrh74/ssDNA	Cardiac/skeletal muscle	P, E	FDA approved	5
LX2006	Friedreich's ataxia	AAVrh74/ssDNA	Cardiac/skeletal muscle	P, E	Phase I/II (NCT05445323)	6
AAV9‐GLB1	GM1 gangliosidosis	AAV9/ssDNA	CNS	P, E	Phase I/II (NCT03952637)	7
SAR446268	Myotonic dystrophy type 1	AAV.SAN011/ssDNA	Skeletal muscle	P, E	Phase I/II (NCT06844214)	7
SGT‐003	DMD	AAV‐SLB101/ssDNA	Cardiac/skeletal muscle	Active (A)	Phase III (NCT07160634)	8
GC301	Pompe's disease	AAV9/ssDNA	Cardiac/skeletal muscle, CNS	P/E	Phase I/II (NCT06391736)	9
INT2104	B‐cell malignancies	LV/ssRNA	Central/peripheral lymphoids	A	Phase I (NCT06539338)	10
UB‐VV400	B‐cell malignancies	LV/ssRNA	Central/peripheral lymphoids	A	Phase I (NCT06743503)	11
Patisiran (Onpattro)	Transthyretin amyloidosis	LNP/siRNA	Liver	E	FDA approved	12
Nexiguran ziclumeran (NTLA‐2001)	Transthyretin amyloidosis	LNP/Cas9 mRNA, sgRNA	Liver	E	Phase III (NCT06672237)	13, 14
NTLA‐2002	Hereditary angioedema	LNP/Cas9 mRNA, sgRNA	Liver	E	Phase III (NCT06634420)	15
DSL101	Wilsons disease	LNP/mRNA	Liver	E	Phase I (NCT07240896)	16
BEAM‐302	Alpha‐1‐antitrypsin deficiency	LNP/adenine base editor mRNA, sgRNA	Liver	E	Phase I/II (NCT06389877)	17
MTS105	Advanced heptocellular carcinoma	LNP/mRNA	Liver	E	Phase I (NCT06689540)	18
MT‐302	Metastatic epithelial tumors	LNP/CAR mRNA	Central, peripheral Lymphoids	A (mRNA enabled)	Phase I (NCT05969041)	19, 20
CPTX2309	B‐cell‐mediated autoimmune disorders	LNP/CAR mRNA	Central, peripheral lymphoids	A (antibody mediated)	Phase I (NCT06917742)	21
Verve‐102	HeFH, PCAD	GalNac‐LNP/Cas9 mRNA, sgRNA	Liver	A	Phase I (NCT06164730)	22
Quaratusugene ozeplasmid	Non‐small cell lung cancer	LNP/plasmid DNA	Lungs	P (charge)	Phase I/II (NCT04486833)	23

As an alternative to viral gene therapies, many groups are pursuing development of tissue‐targeted gene therapies using non‐viral delivery systems. Of this class, lipid nanoparticles (LNPs) are now widely considered the gold standard for non‐viral gene delivery due to the advent of the messenger ribonucleic acid (mRNA) COVID‐19 vaccines.^29^ LNPs are also in clinical use for organ targeted nucleic acid delivery with the approval of ONPATTRO for hereditary transthyretin amyloidosis in 2018.^30^ While LNPs are proven nucleic acid couriers, it is estimated that an approximately 1000‐fold improvement in LNP‐mediated mRNA expression will be needed to render them effective for protein replacement gene therapy applications.^31^ This can be in part achieved by developing more efficient LNPs with greater transfection efficiency for enhanced protein expression. The biodistribution of LNP formulations can also be further optimized to enable highly targeted delivery with a majority of the administered dose directed to the organ/tissue/cell of interest, hence reducing required overall dosage and off‐target effects. As will be elaborated on in our review, polymeric nanoparticles (PNPs) are another important class of non‐viral gene delivery systems under development to address the challenges faced by AAV and LNP. However, to date, PNPs have not achieved broad clinical success for organ targeted nucleic delivery, attributed at least in part to the inherent toxicity of many non‐biodegradable cationic polymer systems and sub‐optimal gene transfer efficiency.^4^, ^32^

It is unlikely our field will find one universally optimal platform for all therapeutic indications which require a tissue‐specific gene delivery approach. As such, we have selected three gene delivery platforms to cover in detail in this review including AAV, LNP, and PNPs. We recognize there are several other established and emerging viral and non‐viral platforms but have chosen these three given their demonstrated modularity, enabling design of tissue‐specific gene vectors for a range of applications. We also recognize for AAV as well as non‐viral DNA‐mediated gene delivery that tissue‐specific promoters can enable selective gene expression in sites of interest. Our attention in this review will primarily focus on viral and synthetic particle biodistributions to target tissues, while additional insight on tissue‐specific gene regulation approaches can be found in these prior reviews.^33^, ^34^, ^35^, ^36^ We also note we have intentionally limited the scope of this review to systemic administration, and readers are directed to these reviews on other local administration modes such as inhaled^37^, ^38^ and intraocular delivery.^39^, ^40^ While clinically meaningful outcomes have been achieved using liver‐targeted *N‐*acetyl‐D‐galactosamine (GalNAc) conjugated short interfering RNA (siRNA),^41^ we note these are all delivered via the subcutaneous route and, as such, this platform will not be discussed here. However, we will discuss in our review the use of GalNAc as an active targeting ligand for nanocarriers delivered via intravenous injection.

Considering the systemic route, we will introduce physiological barriers to organ‐specific gene delivery in tissues such as the liver, lung, lymph node, spleen, and central nervous system (CNS) as well as other difficult‐to‐access tissues such as bone marrow, heart, and placenta. We will describe three mechanisms for organ‐specific targeting of gene delivery systems: passive, active, or endogenous targeting.^42^ Passive targeting refers to how the size, shape, deformability, charge, or other physical features of the gene vector dictate its circulation time and organ accumulation. Endogenous targeting is mainly dictated by the surface composition of the delivery system which governs the composition and conformation of the biomolecular corona that assembles on the particle surface upon exposure to blood or other biofluids, the identity of which can later direct it toward its organ, tissue, or cell‐specific receptors. Active targeting is mediated by the surface functionality of the gene vector of interest, with unique molecules that interact with discrete receptors or features on the target cell. We will also discuss recent literature on the engineering of viral and non‐viral gene vectors that leverage these mechanisms to drive gene vector accumulation to target sites as well as describe the methods to identify such strategies to enhance organ‐specific targeting. As you may appreciate, the literature continues to rapidly evolve with new advances in organ‐specific gene delivery regularly reported. We hope in this review to highlight general principles across these platforms to enable tissue‐specific gene therapies that attain high potency and desirable safety profiles.

## 2 ORGAN‐SPECIFIC BARRIERS

### 2.1 Blood

**FIGURE 1:**
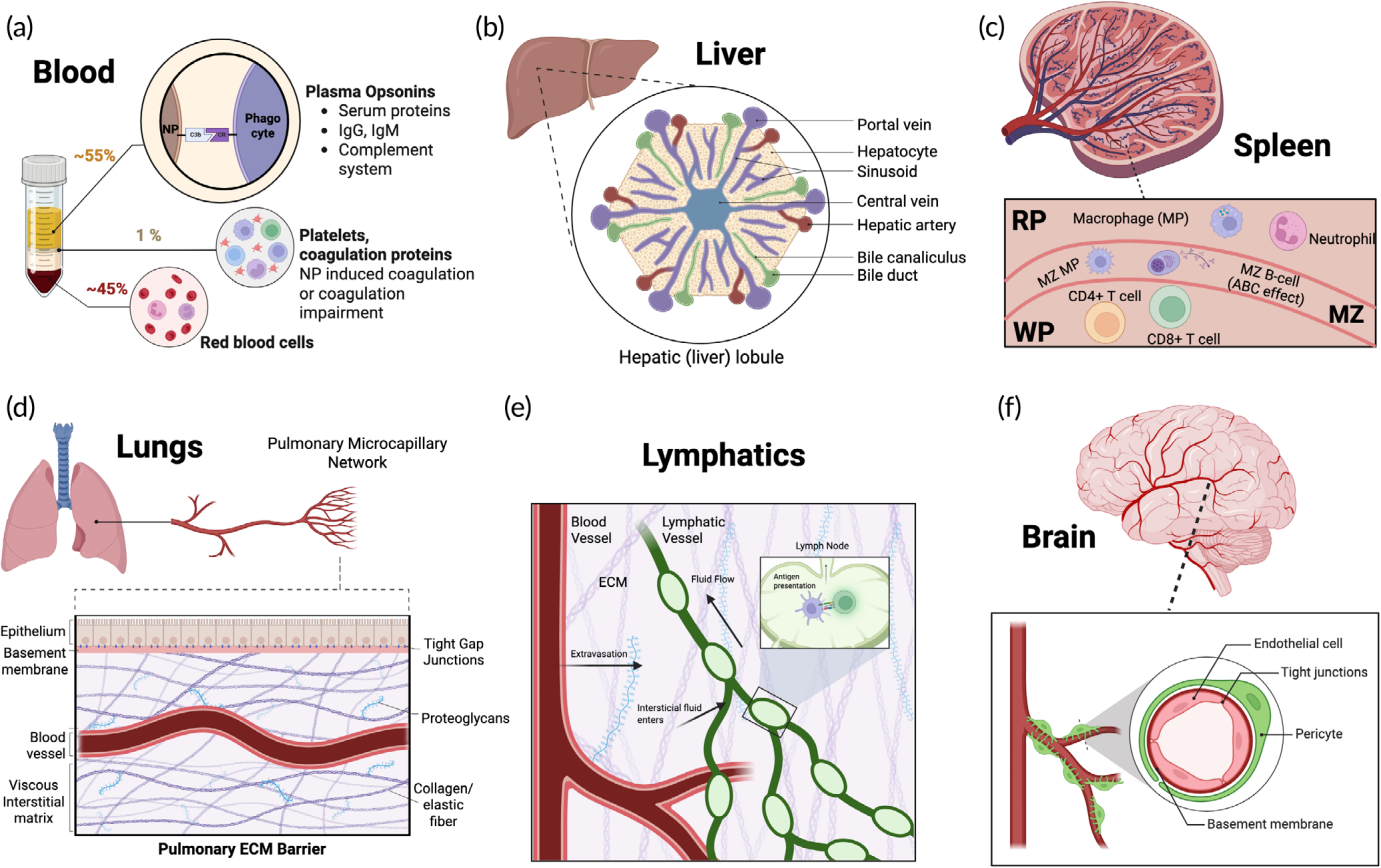
Anatomical and histological features of select biological barriers to gene delivery including (a) blood, (b) liver, and (c) spleen, (d) lungs, (e) lymphatic vasculature, and (f) brain. Created in BioRender. ECM, extracellular matrix; MZ, marginal zone; NP, nanoparticle; RP, red pulp; WP, white pulp.

Upon intravenous (IV) administration, viral and non‐viral gene vectors face the immediate barrier of the blood, where various components (i.e., plasma proteins, lipoprotein, red blood cells [RBCs], platelets) pose a challenge to particle integrity and targeting potential (Figure 1a). Most notably, gene vectors are liable to the absorption of opsonins onto their surface.^43^, ^44^ Opsonins mainly include serum proteins (laminin, fibronectin, collagen type 1, and C‐reactive protein), IgG, IgM, and C3 complement protein fragments. For non‐viral systems, it has been shown that physicochemical properties of the NPs, such as size, shape, and surface composition, largely influence the identity of the opsonizing proteins and the structure of the NP's protein corona which in turn dictates the NP's fate in the bloodstream.^45^ Macrophages in the liver, spleen, and bone marrow typically take up opsonized particles. While substantially more work has been conducted analyzing the protein corona and its impact on NP‐based delivery systems, it should be noted specific serum‐derived components have been shown to accumulate on AAV vectors leading to altered in vivo biodistribution and transduction efficiency.^46^, ^47^

The complement system, comprising 30 plasma proteins, plays a crucial role in opsonization through three activation pathways. All three pathways activate the complement system through the formation of C3 convertase that cleaves C3 into its active forms: C3a responsible for anaphylatoxin, chemoattraction, and inflammation and C3b responsible for opsonization and mediating phagocytosis.^48^ Certain NP properties contribute to complement activation. NPs between 40 and 250 nm can generally activate the complement system. Sizes above 600 nm trigger the complement system to a lesser extent when normalized to their surface area, whereas sizes <30 nm do not activate the complement system.^49^ Hydrophobic and strongly charged particle surfaces are more liable to opsonization and complement activation compared to hydrophilic/neutral counterparts. Certain measures can reduce opsonization, such as grafting of polyethylene glycol (PEG) or certain polysaccharides like chitosan, heparin, or hyaluronic acid. Tolerogenic signals such as CD47 may also be conjugated to the surface of gene vectors to prevent recognition and clearance by macrophages.^43^ One may also alternatively consider designing particles to promote the adsorption of dysopsonins instead of opsonins.^50^, ^51^ Dysopsonins, like histidine‐rich glycoprotein and human serum albumin, are components which block particle uptake by phagocytes.^52^ In the absence of such measures, a conventional NP delivery system would be rapidly tagged with C3b which leads to its immediate elimination from the circulation by macrophages and monocytes of the mononuclear phagocyte system (MPS) within ~10 min of administration.^45^, ^53^

Several past studies investigated NP interactions with the coagulation system which are described in detail in this previous review.^19^ To summarize in brief, these interactions may occur (i) between NPs and cellular components such as platelets, endothelial cells and monocytes, (ii) via coagulation proteins either by adsorption of coagulation proteins (e.g., fibrinogen) making them less available to perform their function in the coagulation cascade, or (iii) direct activation of certain coagulation factors upon contact with anionic NPs such as factor XII.^20^ Surface charge is also a significant factor in NP clearance via coagulation system interaction. However, the nature and fate of charged NPs may greatly vary depending on the particle composition and this has been thoroughly reviewed elsewhere.^45^, ^54^, ^55^

Historically, there is great caution regarding the hemocompatibility of NP gene vectors. Many NP systems have been shown to negatively interact with RBCs resulting in membrane disruptions, cytoskeleton deformation, and oxidative stress as well as aggregation of erythrocytes.^56^ Hence assessing the hemocompatibility of any delivery system is necessary to exclude such undesirable effects. However, more recently, RBCs have also been used as a means of targeting rather than an obstacle through the development of RBC‐hitchhiking of PNPs^57^, ^58^, ^59^ and more recently, this concept has been extended to AAV.^60^

### 2.2 Liver

The liver's physiological role as a filtering organ lends to its role as a major site of accumulation for systemically administered viral and non‐viral gene vectors. The liver is functionally responsible for the sequestration and clearance of metabolic waste and foreign bodies, in addition to synthesizing many functional systemic proteins. The architecture of the liver features liver acini that drain hepatic veins to the sinusoids (Figure 1b). The sinusoidal lumen houses the liver macrophages (Kupffer cells) and is lined by the liver sinusoidal endothelial cells (LSECs). The fenestrae of LSECs are open on the space of Disse, which contains dendritic cells (DCs) and hepatostellate cells (HSC).^61^ Except for gene vectors less than 6 nm or greater than 200 nm in diameter, which are more likely to be cleared by the kidney or spleen, respectively, viral and synthetic particles will accumulate in the liver and hence readily access the liver acini through the sinusoidal lumen. Once inside the sinusoids, gene vectors may encounter the resident Kupffer cells which readily phagocytose particles between 100 and 200 nm in diameter.^62^ AAV (~25 nm in diameter) and synthetic particles less than 100 nm in diameter extravasate through LSEC fenestrations to interact with DCs, HSC, and hepatocytes.^63^

The liver's functional filtration and protein production make it the most widely targeted organ in the clinic. Beyond the passive accumulation of particles in the 100–200 nm size range, many gene vectors experience endogenous targeting to the liver by adsorption of a protein corona enriched with ApoE lipoprotein.^64^ ApoE‐coated particles experience preferential uptake within hepatocytes via low density lipoprotein receptors (LDLR). It has also been previously reported that AAV serotype 8 (AAV8) has a greater capacity for transduction of liver hepatocytes as compared to AAV2 due to targeting via the 37/67 kDa laminin receptor (LamR).^65^, ^66^ Recent evidence also suggests this hepatocyte targeting of AAV8 may be enhanced via binding of serum proteins such as ApoB lipoprotein, human serum albumin, and transferrin without interfering with active targeting via LamR.^67^ Liver‐targeted protein replacement gene therapies have proven a valuable long‐term treatment option for various disorders characterized by defective or absent protein expression in liver hepatocytes. These factors have led to the large clinical presence of liver‐targeted gene therapies (Table 1).^68^, ^69^

### 2.3 Spleen

The spleen is a major secondary lymphoid organ that functions as a site of hematopoiesis and for the filtration of bloodborne pathogens and abnormal cells.^70^ Beyond addressing spleen‐related disorders, the spleen has garnered significant attention in targeted gene delivery for immunomodulation and development of more efficacious vaccines.^71^, ^72^, ^73^ Considering that the spleen serves as a blood filter, systemic IV injection marks the spleen as a significant target for gene therapy. Systemically administered gene vectors within the size range of 200–500 nm have an increased likelihood of uptake by the spleen.^74^ The spleen's venous sinusoid reticuloendothelial cell gap has a similarly sized 200–500 nm range, allowing for the passage of synthetic NPs.^75^ Particles less than 200 nm are more likely subject to uptake by the kidneys and liver.^76^ However, simply modulating particle size to a range of 200–500 nm does not promote efficient spleen targeting. As AAV lies outside of the passive targeting size range, AAV are not expected to readily accumulate in the spleen. However, transduction with AAV8 in splenic B‐cells, T‐cells, macrophage, and dendritic cells has been recently reported using improved in vivo screening methods.^77^

An important consideration for NP gene delivery targeted to the spleen is the need to overcome the splenic barrier (Figure 1c). Located at the spleen's venous sinuses is the red pulp (RP) region of its parenchyma, where blood interacts directly with the spleen.^70^ The RP houses innate immune cells that serve primarily as phagocytic cells. Surrounding larger blood vessels and extending beyond the RP is the white pulp (WP) region that contains the resident lymphocytes. The two pulp regions are separated by a marginal zone (MZ) primarily housing MZ macrophages and MZ B‐cells. The cells of the MZ are thought to play an important role in accelerated blood clearance (ABC). ABC describes the phenomenon where repeated doses of NPs have decreased half‐life in the blood due to increased RES clearance.^78^ The MZ cells can initiate this response through the production of NP‐specific IgM. To efficiently stimulate lymphocytes within the spleen, NPs need to avoid phagocytosis by the reticuloendothelial cells in the RP region. When targeting other organs, LNP and PNP should be engineered to avoid MZ cells to prohibit ABC of subsequent doses.

### 2.4 Lung

The lung is a major port of entry into the body and represents a key target for gene delivery systems used in acute and chronic lung disease applications.^79^ The structure of the respiratory tract consists of a network of airways ending at alveoli that are separated from vasculature by a layer of epithelial cells, an extensive capillary network, and a layer of endothelial cells (Figure 1d). The lung endothelium can be readily accessed and is one of the first destinations for intravenously administered gene delivery systems.^76^ As such, systemic delivery to the lung is also desirable to avoid the challenges associated with the inhaled administration route where gene vectors may be rapidly cleared through airway clearance mechanisms.^38^ However, there are many important barriers the lung presents to achieve efficient penetration of the lung endothelial barrier following systemic delivery. Pulmonary capillaries are 5–10 μm in size and are uniquely tortuous, causing there to be a hindrance in flow dynamics that reduce nanocarrier‐endothelium interactions.^80^ Additionally, the presence of the lung glycocalyx, a specialized section of the extracellular matrix (ECM) comprised of proteoglycans and macromolecules that lies between the endothelial layer and blood vessels, acts as both an electrostatic and steric barrier.^81^ Thus, there are many important considerations in gene vector design that must be accounted for to achieve efficient penetration of the lung endothelial barrier following systemic delivery. Specifically, gene vector size and charge determine its ability to circumvent MPS and arrive at its targeted endothelial destination. For example, particles in the micrometer range tend to accumulate in the lungs due to retention in pulmonary capillaries.^76^, ^82^ However, particles in this size range are unlikely to bypass the highly restrictive tight junctions and/or be uptaken efficiently in pulmonary endothelium. Considering this, past work has endeavored to generate NP systems which aggregate in the pulmonary capillaries and reversibly dissociate to enable efficient uptake into the lung.^83^, ^84^ Past studies have also observed enhanced accumulation and targeting of positively charged gene vectors in the lung following systemic administration.^85^, ^86^ As many PNPs and LNPs possess highly cationic species for effective nucleic acid compaction, many of these delivery systems possess a net positive surface charge which facilitates pulmonary accumulation. However, care must be taken to avoid off‐target effects such as thrombosis and clotting in the pulmonary vasculature following delivery of positively charged lung‐tropic gene vectors.^87^

### 2.5 Lymphatic vasculature

The main function of the lymphatic system is to maintain fluid and immune homeostasis by draining and filtering fluids, macromolecules, and immune cells from peripheral tissue through the lymph nodes and then back into blood (Figure 1e). The lymph nodes are hubs for lymphocytes where presentation of antigens from antigen‐presenting cells (APCs) to CD4+ and CD8+ T‐cell drives the humoral and cellular arms of adaptive immunity, respectively, resulting in the generation of antigen‐specific antibodies and cytotoxic T‐cells.^88^, ^89^ As such, a key target for immunotherapies is the lymphatic vasculature; specifically important are interactions with vaccines and cancer immunotherapies and the immune cells in the lymph nodes.^90^, ^91^ Following systemic injection, therapies must pass critical barriers for effective lymph node targeting.^92^ The primary barrier is the extravasation of NPs from blood vessels into the interstitial space. Here, NPs join the interstitial fluid and encounter the ECM, potentially becoming trapped due to steric or electrostatic interactions with the ECM mesh. Therefore, the size and charge of the NPs must be finely tuned for lymphatic targeting.^93^ Lymphatic fluid, or lymph, is formed when interstitial fluid enters the initial lymphatic vessels due to a pressure gradient between local fluid and the lumen of vessels. When the local fluid's pressure exceeds that of the lymphatic vessel lumen, discontinuous junctions termed button junctions are opened, allowing fluid influx into the lymphatic system. Deformable NPs of larger sizes also can pass through button junctions in the lymphatic endothelium.^94^ For optimal characteristics, particles with a diameter of less than 100 nm and low rigidity provide the best lymphatic transport profiles.^95^, ^96^ More recent work from McCright et al. has also shown densely PEGylated NPs efficiently penetrate lymphatic endothelium through both paracellular and transcellular pathways and accumulate in the lymph nodes to a greater extent than unmodified NPs.^97^, ^98^ Along with paracellular pathways, NPs can enter lymphatics transcellularly through transcytosis mechanisms or trafficking within mature dendritic cells, with their size and surface characteristics dictating these interactions.^99^ Once in the lymphatic system, viral and non‐viral gene vectors would inevitably interact with lymph nodes due to the unidirectional flow.

### 2.6 Brain

Drug delivery through the blood brain barrier (BBB) remains one of the most challenging endeavors in the field of targeted drug and gene delivery. Crossing the BBB is necessary to non‐invasively access the CNS for the treatment of brain diseases. However, the BBB is highly selective to protect the CNS and prevent the entry of unwanted elements into the brain tissue. The blood vessels in the brain are surrounded by a tightly controlled network of endothelial cells that create a physical barrier with tight junctions that prevent the passage of particulates and other unwanted substances (Figure 1f).^100^ The tight junctions of the BBB are uniquely impermeable. There is no free‐flowing passage of large molecules due to open gaps, as is the case in many other physiological environments within the body. Instead, the size, charge, and molecular weight (MW) of the molecules are the predominant factors governing paracellular transport across the BBB. Lipophilic drugs with a MW of less than 500 Da have been shown to passively transport through brain endothelial tight junctions.^101^, ^102^ As it pertains to the movement of substances transcellularly, specific transporters are present on the endothelial membranes and facilitate the movement of certain molecules. For example, glucose and amino acid transporters are present to allow the facilitated transport. Many nanocarriers are not effectively recognized by transporters, and thus, are unable to bypass the BBB. If carriers do make it across, the presence of efflux pumps, which actively transport molecules out of the blood vessels in the brain and back into the bloodstream, works to expel drugs or their carriers before they can reach their target. We will expand currently available carrier‐mediated strategies to cross the BBB via endogenous and active targeting mechanisms in Sections 3 and 4, respectively.

### 2.7 Other difficult‐to‐access organ systems

Several organ systems, including the heart, kidney, placenta, and bone marrow, have proven challenging to achieve tissue‐specific gene delivery using existing platforms. Targeted delivery to the heart requires that gene vectors navigate through a combination of biological and immunological barriers as well as strong mechanical flow, all of which make it a difficult‐to‐access organ for gene therapy. To date, no gene therapies have been approved for cardiac diseases, such as cardiomyopathies,^103^ which may be due in part to low targeting efficiency to cardiac tissues, specifically the myocardium that is separated from blood by the endothelial barrier.^104^ Targeted cardiac delivery requires NPs that can avoid MPS with strong binding to the cardiac endothelium.^105^ This has led to preclinical development of active targeting strategies for cardiac targeting^106^ and the exploration of other delivery routes such as intracoronary delivery.^107^ As compared to other naturally occurring AAV serotypes, AAV8 has been shown in a previous study to mediate the highest level of gene expression in the heart following IV delivery.^108^

The kidneys are another difficult‐to‐access organ due to the glomerular filtration barrier (GFB) and off‐targeting to the liver. The GFB is comprised of multiple layers with varying pore sizes, including the glomerular epithelium (70–100 nm), basement membrane (4–10 nm), and filtration slit diaphragms (35–45 nm) as described in a recent review.^109^ As such, NPs with a diameter of 10 nm can pass through the GFB and accumulate in the kidneys, although to a lesser extent than the liver.^110^ This size restriction significantly limits the type of NPs able to access the kidney and the size of their gene cargo. Charge also plays a role in kidney targeting as the GFB possesses a highly negative net charge. Consequently, NP disassembly may occur at the GFB, leading to cargo filtering into urine.^111^ Additionally, NPs in the conventional range of 100–200 nm tend to be filtered and accumulate in the liver as stated above. These barriers have limited the clinical success of systemic gene delivery targeting the kidneys thus far.

The placental barrier offers a unique challenge for drug delivery. Two distinct layers surround the fetal capillaries: syncytiotrophoblast and cytotrophoblast. With advancing gestation, these layers progressively thin, and the cytotrophoblast layer is almost entirely lost by term. The partial pressure gradient of gasses such as oxygen facilitates diffusion into fetal arteries through the cytotrophoblast layers. Throughout term, drugs with a MW of less than 500 Da diffuse through the placenta via passive diffusion.^112^ Above 500 Da, drug diffusion across the placenta is dependent on molecular conformation or must rely on carrier‐mediated transport.^113^ Conversely, NPs have been used to encapsulate drugs and prevent them from crossing the placental barrier (i.e., preventing fetal drug exposure). It is clear that size, charge, and rigidity play an important role in a NP's ability to traverse the placenta yet defined effects of these physical properties have not been fully established.^114^ However, more recent work from the lab of Michael Mitchell has shown LNP with enhanced stiffness (by increasing content of the helped lipid β‐sitosterol) are more readily uptaken into the placenta and exhibit reduced liver accumulation.^115^ Using a protein‐corona‐mediated endogenous targeting approach, Swingle and colleagues were able to demonstrate effective delivery of vascular endothelial growth factor (VEGF) mRNA LNP to the placenta for the prevention of preeclampsia in a murine model.^116^ Other active and/or endogenous targeting strategies to modulate gene vector internalization in specific cell types or binding to surface receptors upregulated in placental tissue are also being explored to enhance gene delivery to this site.

The bone marrow is another difficult organ to target. Bone marrow is located in the centers of long and axial bones. It functions as the primary hematopoietic organ and a primary lymphoid tissue, producing erythrocytes, granulocytes, monocytes, lymphocytes, and platelets. Many of these hematopoietic cells present in the bone marrow are great candidates for gene therapy, but delivery of these therapies is challenging due to the presence of the bone marrow barrier (BMB).^117^ The BMB consists of specific vascular structures, namely the sinusoidal epithelium, which tightly controls the entry and exit of cells and molecules between systemic circulation and the bone marrow. These structures prevent pathogens and toxins from entering the bone marrow to preserve and support hematopoiesis^118^ and have an estimated maximum pore size of about 61 nm, which can also limit the transport of nanocarriers.^119^ Additionally, certain features of hematopoietic cells further contribute to delivery challenges. For example, hematopoietic stem cells (HSCs) are extremely rare, must be edited without altering their stem cell properties, and are difficult to transfect. For these reasons, ex vivo gene editing is the gold standard method for editing HSCs, and a number of hematopoietic stem cell gene therapies have been implemented in the clinic.^120^, ^121^, ^122^ However, this method has drawbacks, and effective in vivo delivery solutions are being explored. One promising study has shown that surface enrichment of apolipoprotein E on LNPs gave them bone marrow targeting ability and facilitated delivery to a variety of bone marrow cells.^123^

## 3 GENE DELIVERY PLATFORMS

### 3.1 Adeno‐associated virus

#### 3.1.1 Structure and function of AAV

AAV is a naturally occurring member of the Dependoparvovirus genus.^124^ It is a replication‐deficient virus, relying on the presence of a helper virus (adenovirus) to infect humans, non‐human primates, and other mammalian species. AAV is a non‐enveloped virus comprised of a capsid protein shell that houses the genetic material within. The capsid is composed of three integral viral proteins (VPs) that assemble into an icosahedral symmetry structure. The AAV capsid proteins, VP1, VP2, and VP3, are present in a 1:1:10 ratio within the capsid, respectively.^124^, ^125^ VP1 is the largest among the capsid proteins, roughly 87 kDa in size, and plays a key role in the ability of AAV to enter the host cell. VP2 is approximately 73 kDa where prior work has shown this capsid subunit is dispensable and an ideal target for modifications with non‐native peptide (e.g., targeting moieties).^126^ VP3 is the smallest of the three capsid proteins, approximately 62 kDa, but is the most abundant of the three and is critical for viral capsid assembly. As such, VP3 is necessary to maintain the overall integrity of the capsid. These three capsid proteins assemble 60 protein subunits to form a highly ordered icosahedral capsid, forming a capsid that is roughly 20 nanometers in diameter.^27^, ^127^ More recent literature also suggests AAV capsid proteins are involved in episomal gene expression suggesting these elements facilitate both viral entry in target cells and persistence of gene expression.^128^, ^129^ As such, the functional properties of AAV capsids dictate many key features necessary for their therapeutic use and have been the focal point of AAV engineering for tissue‐specific gene delivery.

AAV contains a single‐stranded DNA genome of ~4.7 kilobases.^26^, ^130^ There are three main components to the AAV genome: the inverted terminal repeats (ITRs), the replication (*rep*), and capsid (*cap*) genes. ITRs are sequences that are crucial for the replication and packaging of the virus and are located at both ends of the genome itself.^131^ Once the virus transduces the host cell, the ITRs serve as a signal for intracellular production of AAV (e.g., rescue, replication, and packaging) and for most recombinant AAV are derived from AAV serotype 2. The *rep* gene encodes for the proteins utilized in AAV viral genome replication and integration into the host cell genome. The *rep* gene encodes proteins essential for viral propagation and host transduction. The *cap* gene encodes for the capsid proteins (VP1, VP2, and VP3). An alternate open reading frame in the *cap* gene encodes for the assembly‐activating protein (AAP) which aids in the structural integrity of the capsid during assembly.^132^ Recombinant forms of AAV used in gene therapy applications are generated by replacing *rep* and *cap* genes with a transgene of interest.

To initiate binding to the cell, many AAV interact with several cell‐surface associated glycans and proteoglycans terminated with galactose, sialic acid, and heparan sulfate.^133^, ^134^ Protein co‐receptors such as FGF receptors, 37 kDa/67 kDa LamR, or specific integrins also play a large role in cell binding, contingent upon the AAV serotype and tissue type.^134^ AAV entry into host cells is highly regulated and for many AAV serotypes requires interactions with the AAV receptor (AAVR)—the transmembrane protein KIAA0319L.^135^ Once AAV has bound to AAVR and/or other co‐receptors, the virus enters the cells via receptor‐mediated endocytosis. Upon entry, the acidic environment of the endosome triggers a conformational change in the capsid proteins that allows a facilitated release of the viral genome into the cytoplasm.^136^ The host cell machinery is then used to facilitate transcription, replication, and integration of the AAV genome into the host genome.

#### 3.1.2 Immunological barriers to AAV gene delivery

AAV immunogenicity, defined as the propensity of the gene vector to evoke an immune response by the host, has been studied extensively as this could render these therapies ineffective if not appropriately controlled. While generally considered less immunogenic compared to other viral vectors, this response is dependent on the AAV serotype used, dosage requirements, and tissue type being targeted.^137^, ^138^ AAV vectors can trigger innate immune responses through the recognition of the AAV genome via pattern recognition receptors. The pattern recognition receptors can trigger a mild immune response, including natural killer cell activation or interferon production. Zhu et al. demonstrated that the TLR9‐MyD88‐type I interferon (IFN) pathway played a major role in the immune response to AAV, and that inhibiting this pathway had the potential to improve the efficacy of AAV.^139^ In addition, Hösel et al. observed the innate immune response to AAV vectors in human liver cells.^140^ Their work found that the vectors were recognized by Toll‐like receptor 2, which in turn caused an upregulation of inflammatory cytokines, initiating a larger immune response. Recent research conducted by Bucher et al. found that the innate immune responses brought on by AAV in dendritic cells could be attributed to extra unpackaged viral DNA found in AAV vectors during preparation, revealing potential pathways to mitigate the immune response seen by improving the AAV vector purification process.^141^ AAV vectors can also induce an adaptive immune response over time, specifically with repeated serotype use or tissue targeting. These subsequent administrations can lead to the production of neutralizing antibodies against the capsid proteins, which in turn can neutralize the virus and reduce its effectiveness. In short, the immunogenicity of AAV is very dose dependent, where less frequent (e.g., staggered over months) and lower dosages result in a weaker immune response.^142^, ^143^ An epidemiology study conducted by Calcedo et al. found that neutralizing antibodies affected different AAV serotypes to varying degrees, and that the presence of neutralizing antibodies greatly hindered AAV‐mediated transduction.^144^ Further, it was found that AAV2 is more likely to provoke a strong immune response and the antibodies to AAV2 were most common, followed by antibodies to AAV1, in comparison to other AAV serotypes, such as AAV7 and AAV8.

Although AAV is considered non‐pathogenic and safe for clinical use, large doses can cause immunotoxicity (e.g., cytokine storm) in tissues such as the liver, lung, and heart which can lead to potentially severe and life‐threatening outcomes.^145^ Hinderer et al. conducted a study wherein non‐human primates and piglets displayed severe toxicity following IV injection of AAV vectors.^146^ Their study concluded high AAV doses lead to both systemic and sensory neuron toxicity, regardless of the transgene or serotype used. Another safety concern for insertional mutagenesis arises when the viral genome integrates into the host's genome, which can lead to oncogenesis or genetic abnormalities. Other gene therapies that involve vectors, such as lentiviruses or retroviruses, can fully integrate their genomes into the host DNA, while AAV generally does not readily integrate into the host genome. But in rare cases, often induced by high doses or certain serotypes, AAV can integrate into the host genome at specific chromosomal regions.^136^ An analysis of AAV integration sites within the human chromosome conducted by Miller et al. elucidated the key integration sites and a profile for insertional mutagenesis risks for AAV vectors.^147^ They found that insertional mutagenesis hotspots exist within the chromosome, but subsequent work has shown that AAV vector design can be further optimized to mitigate this risk.^148^

The key challenge in AAV‐based gene therapy is the pre‐existing immunity that exists among the patient population. As AAV infections are naturally occurring, it has been found ~80% of the human population possess anti‐AAV neutralizing antibodies where their presence may hinder broad deployment of AAV gene therapies.^149^ Considering this, patients receiving AAV gene therapies are often treated with corticosteroids to mitigate the pre‐existing antibodies and dampen the immune response. Varying the serotypes used can overcome this pre‐existing immunity, as some serotypes, such as AAV9, may not be as widely encountered in the general population, and thus have a lesser immune response.^150^ As for the long‐term safety of AAV‐based gene therapies, research is still actively being pursued to identify and define the factors that impact long‐term patient health.

#### 3.1.3 Endogenous targeting and biodistribution of AAV

AAV vectors exhibit a range of tissue‐dependent tropisms, determined primarily by the serotype of AAV and the ability of those serotypes to identify and bind to specific cell surface receptors. As such, endogenous and active targeting are not mutually exclusive concepts in the design of AAV for tissue‐specific gene delivery. There are 12 AAV serotypes available and we will highlight a few commonly employed in gene therapy applications. Table 2 summarizes previously identified co‐receptors and tissue tropisms of naturally occurring AAVs. We note while there is generally consensus on serotypes most amenable for transduction in specific tissues (e.g., liver‐tropic serotypes AAV3/AAV8, BBB‐penetrating variants of AAV9). However, comparisons are often difficult across studies given the differences in AAV manufacturing between laboratories as well as selection of reporter transgene and promoters utilized. Moreover, most AAV serotypes show broad activity in a range of tissues with a preference for certain tissues based on the presence and overall levels of their co‐receptors on permissive cell types. As such, we will highlight outcomes of a two key studies with head‐to‐head comparisons of biodistribution for multiple AAV serotypes following IV administration in mice. To the best of our knowledge, similar analyses have not been conducted in non‐human primates or other large animal models.

**TABLE 2:** Summary of co‐receptors and tissue tropism for naturally ocurring adeno‐associated virus (AAV) serotypes 1–12.

Serotype	Known co‐receptor(s)	Primary tissue tropism(s)	References
AAV1	N‐ and O‐linked SiA	Muscle, lung	151, 152, 153, 154
AAV2	HSPG, aVB5 integrin, FGFR1, LamR	Liver, retina, CNS	28, 155
AAV3	HGFR, FGFR1, LamR, HSPG	Liver	156, 157, 158
AAV4	N‐ and O‐linked SiA	Liver, heart, retina, CNS	155, 159
AAV5	N‐ and O‐linked SiA, HSPG	Lung, liver	155, 159
AAV6	N‐ and O‐linked SiA, HSPG, EGFR	Lung, muscle, liver	160, 161
AAV7	Unknown	Liver	162
AAV8	LamR	Liver, muscle, heart, retina	66
AAV9	N‐terminal galactose	CNS, muscle, liver	133
AAV10/AAVrh10	N‐terminal galactose	CNS	163, 164, 165
AAV11	Unknown	CNS	166, 167
AAV12	N‐ and O‐linked SiA, HSPG	Nasal	168, 169

Abbreviations: CNS, central nervous system; EGFR, epidermal growth factor receptor; FGFR1, fibroblast growth factor receptor 1; HGFR, hepatocyte growth factor receptor; HSPG, heparan sulfate proteoglycan; LamR, 37 kDa/67 kDa laminin receptor; SiA, sialic acid.

**FIGURE 2:**
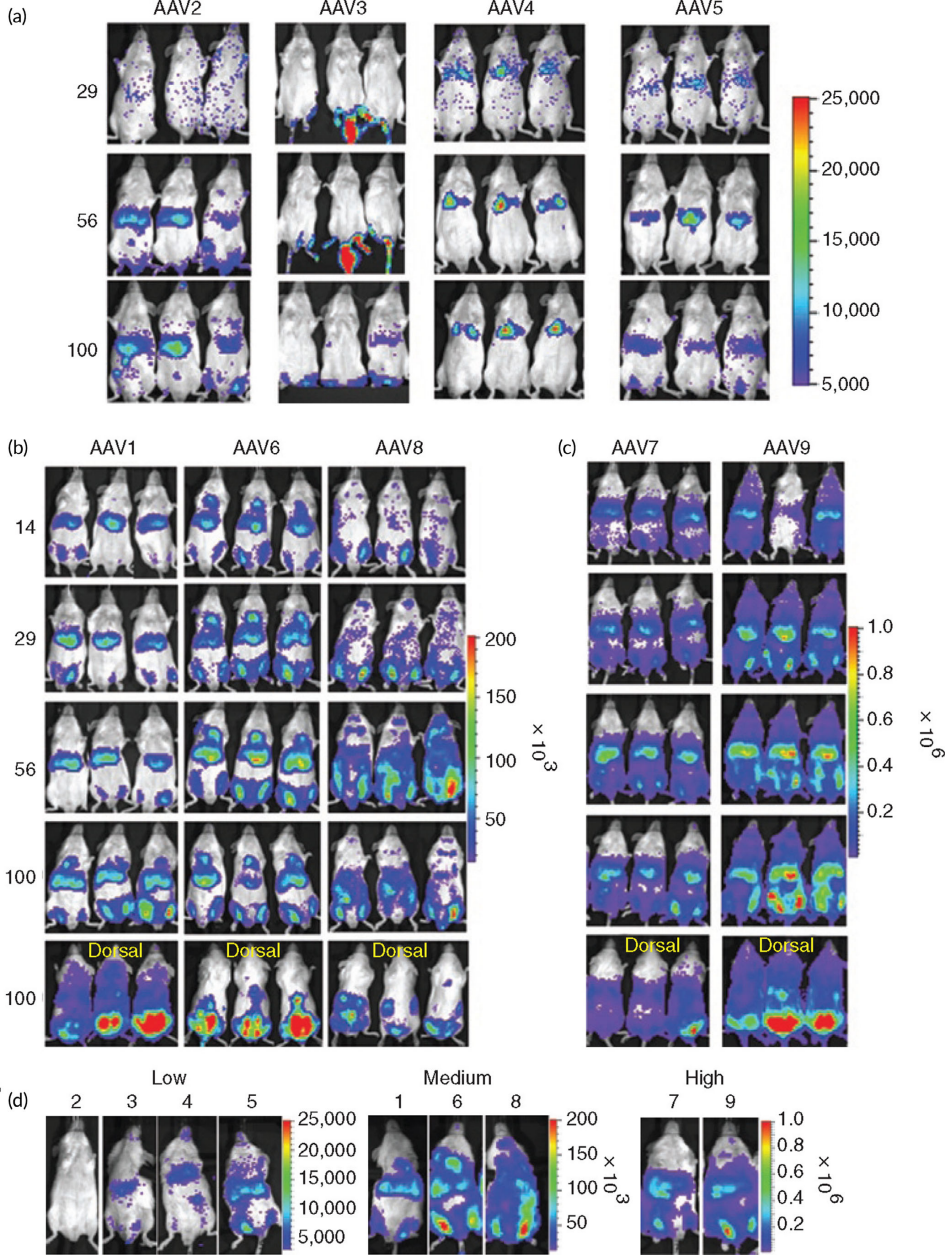
Comparative assessment of transduction performance for adeno‐associated virus (AAV) serotypes 1–9. Following tail vein injections in mice with AAVs packaged with Cytomegalovirus‐Luciferase assessed using IVIS imaging at 14, 29, 56, and 100 days post‐administration: (a) AAV serotypes^2^, ^3^, ^4^, ^24^: Low expression groups (5000–25,000) photons/s/cm^2^. (b) Medium expression (15,000–25,000) photons/s/cm^2^. (c) High expression groups (10,000–1,000,000 photons/s/cm^2^). (d) Luciferase expression 9 months after administration for AAV1‐9. Reproduced with permission from The American Society of Gene and Cell Therapy.

Zincarelli et al. performed a comprehensive assessment of in vivo gene expression and biodistribution of AAV vector serotypes 1–9 in mice^162^ (Figure 2). The study was one of the first in the field to perform direct comparisons of tissue tropism profile for multiple AAV serotypes, produced in the same manner, carrying the same transgene (luciferase) under the control of the same promoter, and across a broad range of tissues following tail vein injection. They found that each serotype had varying levels of expression, and defined these levels as low, moderate, and high expression groups by monitoring bioluminescence via IVIS in live animals. AAV2, AAV3, AAV4, and AAV5 were of the low expression group (Figure 2a). AAV1, AAV6, and AAV8 were of the moderate expression group (Figure 2b). AAV7 and AAV9 were of the high expression group with bioluminescent signal detected primarily in the liver (Figure 2c). In all cases, gene expression persisted for up to 9 months (Figure 2d) highlighting the durability of expression using AAV gene vectors. As for distribution, it was observed that the liver and the hindlimbs were the main location of expression for AAV1, 2, 5, 6, 7, and 9. AAV8 and AAV9 were expressed more ubiquitously throughout tissues with AAV9 showing the strongest transduction overall. Interestingly, they also found that AAV4 had the highest expression in the lung and kidney, with additional expression seen in the heart. AAV6 was also seen to have expression in the heart, as well as in the liver and skeletal muscle. This study demonstrates how differences in AAV capsids across serotypes can significantly affect transduction in tissues throughout the body.

A recent study published by Walkey et al. further examined the tissue and cellular tropisms of multiple AAV serotypes following IV injection in mice.^170^ AAV3B (a subtype of AAV3), AAV4, AAV5, AAV6, AAV7, AAV8, AAV9, AAVrh8 (rhesus macaque‐derived AAV8 variant), AAVrh10 (rhesus macaque‐derived AAV10 variant), and AAVrh74 (variant derived from rhesus macaque muscle tissue) were delivered systemically to male and female mice to observe transgene expression across tissues. The majority of AAV serotypes, except AAV3B and AAV4, displayed substantial liver transduction further highlighting the challenges in achieving extrahepatic gene delivery. They also found that AAV4 was observed in endothelial cells in most tissues, as well as having transduced beta cells in the pancreas. Interestingly, transduction with several AAV serotypes was also observed in the adrenal, testicular, and ovarian tissues which, to the best of our knowledge, have not been sites of transduction documented in prior work.

#### 3.1.4 AAV capsid engineering—design approaches

Researchers often employ directed evolution to optimize AAV capsid properties, enhancing and altering capsid proteins' characteristics to influence the vector's behavior.^27^ Directed evolution is the process of generating a new, distinct amino acid sequence in the capsid and selecting for capsids possessing desirable properties. In the scope of AAV capsid design, directed evolution allows researchers to specifically engineer capsid proteins (VP1, VP2, and VP3) to enhance existing or grant novel capsid characteristics promoting higher transduction efficiency, increased tissue specificity, and increased immune evasion. An efficient strategy to generate a library of novel AAV capsids is error‐prone polymerase chain reaction (PCR), which relies on random mutations' introduction into the gene encoding the AAV capsid proteins during the amplification process. This process aims to introduce such mutations at a high rate, creating a vast library of genetic variants of the capsid. This library is then tested for favorable characteristics that will enhance the transduction efficiency of the AAV capsids. For example, Qian et al. employed error‐prone PCR to engineer AAV5 for applications in hepatocytes.^171^ They could improve the AAV5 transduction efficiency while simultaneously maintaining the vector's uniquely low seroreactivity. The capsids resulting from this study allow for optimized hepatocyte transduction and are a promising option for liver‐targeted gene therapies. Characteristics such as cell binding, capsid formation, and genome packaging are tested and screened to filter for those that show optimal characteristics for the specific application.

Another common method used for AAV library generation is phage display. This method employs genetically modified phages, which are viruses that specifically infect bacterial cells to establish and optimize a capsid library. The phages are genetically engineered to express a broad range of varying capsid proteins on their surface; libraries of these phages are then screened for their affinity to a specific ligand or cell type in a high‐throughput fashion. Capsid variants or proteins that display a high affinity to targets of interest are retained and amplified to improve the desired behavioral components of the capsid. Work et al. employed this method in their early work to develop AAV2 viral vector capsids that were selective to and had enhanced transduction of vascular smooth muscle cells.^172^ By employing specific ligands from the smooth muscle cells that were identified via phage display, the AAV2 vectors were amenable to being engineered to specifically recognize and successfully bind to vascular smooth muscle cells more efficiently. Phage display proves very useful in identifying AAV capsid variants that have superior tropisms in varying tissue, as those capsid variants and proteins that are more favorable for a certain tissue type will display higher affinities to those target cells and target receptors. Additionally, researchers can narrow down AAV capsids based on their immunogenicity and protein interactions by exposing capsid variants to neutralizing antibodies or peptides, then observing those that are able to resist and avoid their immunogenetic effect.

Rational design approaches have also been used to optimize AAV vectors for enhanced tissue specific gene delivery. One approach is to create chimeric or hybrid AAV vectors. Chimeric vectors are formed by combining capsid proteins from varying AAV serotypes to create novel vectors with enhanced characteristics such as reduced immunogenicity or increased tissue tropism.^27^ Early work by Hauck et al. demonstrated the concept in a very functional manner, as they developed a chimeric AAV vector using capsid components from both AAV1 and AAV2 to combine the transduction characteristics of both vectors.^173^ In vivo data demonstrated that their newly formed chimeric vectors displayed liver and muscle transduction that was similar to that of the original parent vectors, hence demonstrating a successful implementation of the concept. Varying combinations can be employed to create new hybrid capsid formations and examine their benefits on gene delivery in a range of specific gene therapies. However, it should be noted that many AAV are closely related and may possess as few as six divergent amino acids in the entirety of their capsid (e.g., AAV1 and AAV6). As such, chimeric vector engineering approaches are best suited for AAV serotypes which are phylogenetically distinct and/or possess unique receptor binding preferences. Further mapping of the AAV capsid and receptor binding domains has also allowed for researchers to rationally design AAVs with enhanced binding affinities for their target cells and/or optimize AAV vector unpackaging once internalized.^174^, ^175^

#### 3.1.5 AAV capsid engineering—screening methods

In vitro screening can be used to efficiently evaluate a library of AAV capsid variants based on their transduction efficiency. This provides researchers the means to readily identify capsid variants with improved delivery capabilities, as well as identifying structural and/or characteristic features useful in transducing specific tissues, binding to specific receptors, crossing certain delivery barriers, or even navigating the immune system.^176^ For example, Schröder et al. generated an AAV library via error‐prone PCR and screened this library in vitro to identify aorta‐tropic AAV variant using vascular smooth muscle cells.^177^ They found that a novel peptide motif (RFTEKPA) improved in vitro transduction efficiency compared to previous motifs and further validated these engineered vectors in an ex vivo aorta model. In vivo screening is another step used to screen libraries of AAV capsid variants, where the vectors are tested directly in animal models to screen for enhancements in tissue delivery and gene expression. This can be accomplished by delivering a library of barcoded AAV to mice such that highly efficient AAV can be subsequently identified in specific tissues via next‐generation sequencing.^178^, ^179^ Screening for vectors that have high transduction efficiency in organs of interest while minimizing off‐target transduction is a key aspect to gene therapy that can help mitigate side effects as well as unwanted immune responses. An important consideration for the in vivo screening is these efforts may yield vectors highly efficient in animal models from which they were derived but less efficient in human tissues due to species‐specific differences in host receptor expression.^180^, ^181^ AAV vectors obtained from screening in non‐human primates typically yield vectors that are effective in humans^182^ but broad screening of AAV in non‐human primates requires significant expense. Alternatively, these concerns can be mitigated, at least in part, with a mixture of screening in both human in vitro models and in vivo animal models.

**FIGURE 3:**
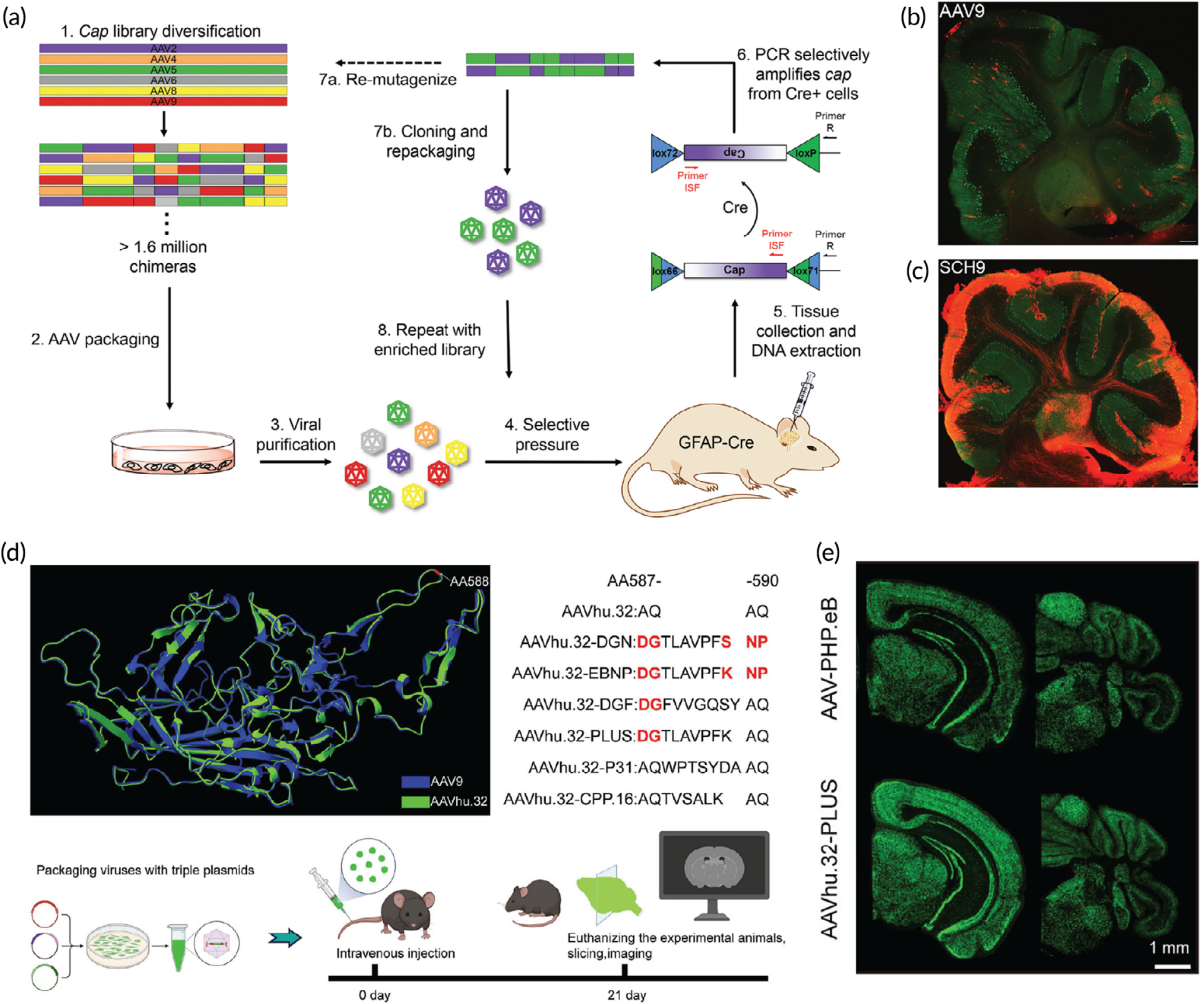
Capsid engineering approaches to generate neurotropic and blood brain barrier‐penetrating adeno‐associated virus (AAV) gene vectors. (a) Graphical depiction of Cre‐dependent in vivo screening approach to evaluate computationally designed AAV library generated using SCHEMA and identify AAV capable of transducing neural stem cells. Transduction (tdTomato expressin) within the cerebellum of mice following intracranial injection of (b) wild type AAV9 and (c) engineered variant of AAV (SCH9) with enhanced neurotropism. The results shown in (a–c) were previously published by Elsevier and reproduced under the terms of the CC BY‐NC‐ND 4.0 open access license. (d) Engineering approach for amino acid optimization in engineered variants AAVhu.32 that are capable of bypassing the blood–brain barrier. (e) Transduction (enhanced green fluorescent protein (eGFP) expression) within the brain of mice following intravenous injection of known BBB‐penetrating AAV‐PHP.eB and newly identified AAVhu.32‐PLUS. The results shown in (d, e) were previously published by the American Society of Gene & Cell Therapy and reproduced under the terms of the CC BY‐NC‐ND 4.0 open access license.

Computational modeling can also be used to further refine capsid structure variation on its function.^183^, ^184^ For example, Ojala and colleagues established a computational model called SCHEMA that mapped protein structure and optimal crossover points of DNA shuffling and used it to create a hybrid AAV capsid library^185^ (Figure 3a). Their SCHEMA‐generated library was functionally diverse and could be analyzed for optimal in vitro properties. SCHEMA was eventually able to generate a novel AAV capsid that efficiently transduced neural stem cells in an in vivo mouse model (Figure 3b,c). Thus, this method allows for a more in‐depth analysis and understanding of the genetic components responsible for the AAV capsid's performance and can help predict the impact of novel genetic sequences or variants before they are synthesized and applied. Through a combination of computational modeling, rational design, and in vivo screening, engineered variants of AAV have also been identified capable of efficiently bypassing the BBB and achieving robust transduction within the CNS following IV administration^186^ (Figure 3d,e). Structural AAV analysis can be conducted to establish links between directed evolution induced structural changes to the AAV capsid and their therapeutic and immunological effects. Cryo‐electron microscopy, x‐ray crystallography, and nuclear magnetic resonance are all methods employed to assess detailed changes in the AAV capsid allowing for further optimization of protein interactions with target cells and the immune system through further rounds of directed evolution.^187^, ^188^

A way to mitigate the immune response is by conducting a final screening step focused on selecting capsid variants against neutralizing antibodies that are found in human sera.^177^ As previously mentioned, the presence of pre‐existing neutralizing antibodies is a major barrier to effective AAV‐mediated gene delivery. A method currently used to select AAV capsid variants resistant to these neutralizing antibodies is screening the AAV library of capsid variants against plasma/serum with those antibodies and assessing and quantifying the immune response.^189^, ^190^ Variants that are least affected and can deliver their cargo are favorable and are selected for, while those that are hindered greatly are selected against. Recently, Chhabra et al. conducted a broad study examining the activity of neutralizing antibodies against six AAV serotypes in a sample of over 500 adults from 10 countries.^191^ They conducted a transduction inhibition assay and established an immune response profile elucidating the relationship between neutralizing antibodies and AAV serotypes, which can prove key in future work in vector design.

### 3.2 Lipid nanoparticles

#### 3.2.1 Structure and function of LNP

LNPs possess many advantages for nucleic acid delivery, including relatively simple and scalable composition and manufacturing,^192^ high encapsulation efficiency, efficient cargo shielding within their aqueous core, and strong transfection capabilities in diverse cell types. The four main components of conventional LNPs are ionizable tertiary amine‐containing lipids, cholesterol, helper lipids, and PEGylated lipids.^193^ The ionizable lipid components are nearly uncharged at physiological pH but progressively acquire a cationic charge as the environmental pH drops.^194^ This feature allows their spontaneous electrostatic coacervation with the anionic RNA during LNP assembly and later following LNP endocytosis can drive efficient endosomal membrane disruption and hence endosomal escape of the intact RNA cargo as the pH of the endolysosomal compartment begins to decrease.^195^

Maintaining colloidal, physicochemical, and functional stability of LNPs in physiological media has proven a significant challenge. For example, it has been shown plasma exposure can accelerate lipid desorption from LNPs as a result of clotting factors which alter their degradation kinetics.^196^ PEG desorption rate is another factor that affects both particle stability in physiological media and organ localization. Rapid desorption of PEGylated lipids leads to greater localization in the lung, liver, or spleen, while slow desorption negatively impacts cellular uptake and endosomal escape.^197^ Meaningful work from Voke et al. introduced a novel method to separate hard protein corona‐associated LNPs from other nanoparticulate plasma components, such as exosomes and lipoproteins, using a combination of density gradient‐based separation and mass spectrometry‐based proteomics.^198^ They later studied the impact of the most abundant proteins in the hard corona of the LNP, either individually or in a mixture, on cellular uptake, endosomal escape, and mRNA transfection in HepG2 cells. They demonstrated that ApoE, despite promoting cellular uptake of LNPs, reduced endosomal escape and compromised mRNA transfection. Similarly, vitronectin, despite increasing cellular uptake, has been shown to reduce transfection efficiency of LNPs.^198^ It should be noted this study primarily focused on uptake in a hepatic cell line where it would be valuable to build on these important findings and further study vitronectin‐dependent uptake in other cell types. Cholesterol content has also been shown to affect the colloidal stability and degradation rate of LNPs upon systemic circulation. Specifically in prior work, increases in cholesterol molar ratio led to improved stability and reduced degradation rate, as well as reduced RNA cargo leakage from the particle.^199^, ^200^

Conventional LNP formulations have been found to accumulate predominantly in the liver through an endogenous targeting mechanism mediated by apolipoprotein E (ApoE). The ionizable lipid component inherently dictates the natural tropism to the liver upon IV administration via ApoE‐mediated endogenous targeting.^201^ In addition to the ApoE‐mediated endogenous targeting,^62^ the natural hepatic tropism of LNPs results from a combination of the structural and functional features of the liver previously described in Section 2.3. Yet, the potential applications of mRNA replacement therapies beyond the liver are vast, and manipulating LNP's structure to shift their natural hepatic tropism toward extrahepatic organs has been widely sought after. An emerging concept is that of selective organ‐targeted LNPs (SORT LNPs), which draws inspiration from the ApoE‐mediated hepatic targeting of currently commercialized LNPs. Siegwart and colleagues have reported that adding a fifth component coined “sorting lipid” to the LNP can influence its protein corona composition, adding novel targeting entities to the LNP surface in situ following IV administration.^202^ This SORT lipid redirects LNP toward specific receptors on specific organs that would not be accessible otherwise.

As discussed in Section 2, particle size can greatly influence a nanocarrier's targeting to and within different organs. Particle size also impacts the glomerular filtration and hence the clearance rate of the particle from the circulation which further impacts the potential for extrahepatic accumulation which increases as a function of circulation time. LNPs are most commonly prepared by solvent injection using microfluidic devices. In addition to being an easily scalable and highly reproducible technique, it also provides better control over the resulting LNPs' size and polydispersity through the careful control of different mixing parameters.^203^ Belliveau et al. previously demonstrated that using staggered herringbone micromixers, LNPs' size and polydispersity were inversely proportional to the total flow rate (TFR) at which fluids were introduced into the device.^204^ They also showed that using higher molar ratios of PEGylated lipids has been shown to reduce the particle size of LNPs. A comprehensive study by Roces et al. also demonstrated similar results regarding the relation between particle size, polydispersity, and TFR, though they demonstrated that the size stabilized at 10–20 mL/min.^205^ In their study, they also demonstrated that both size and polydispersity decreased with increasing aqueous to organic phase flow rate ratio (FRR), with both parameters starting to plateau at an FRR of 3:1. Additionally, they showed that increasing cholesterol while reducing cationic lipid contribution resulted in smaller, more monodisperse particles that showed higher stability following purification and dilution steps, yet such an effect was not observed for ionizable lipids. Okuda et al. showed that adding NaCl to the aqueous buffer led to the formation of larger LNPs in a concentration‐dependent fashion, and that such larger LNPs led to higher transgene expression in DCs in vivo.^206^

**FIGURE 4:**
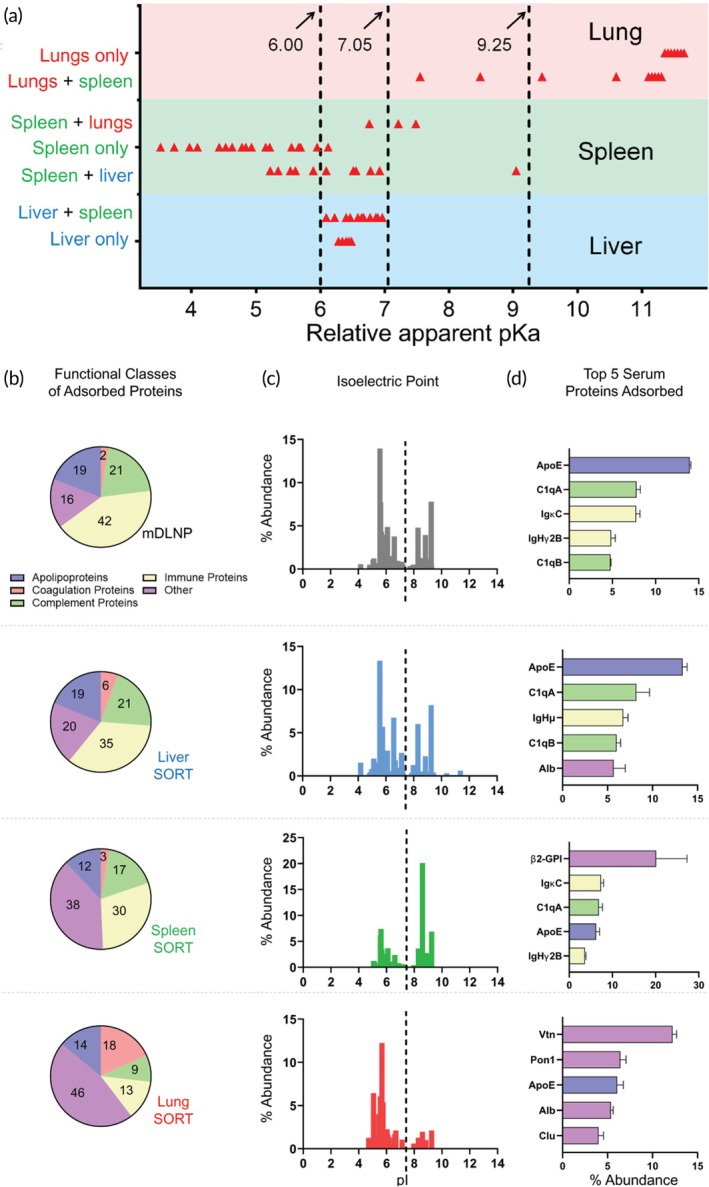
Impact of the apparent pKa of lipid nanoparticles (LNPs) on their organ tropism and endogenous targeting. (a) Varying organ tropism of selective organ targeting (SORT) LNPs with varying their apparent pKa. (b) Functional classes, (c) isoelectric points, and (d) top five most abundant adsorbed protein corona components for liver, spleen, and lung SORT‐LNPs. Reproduced with permission from PNAS. Copyright © 2021 National Academy of Sciences, USA.

Apparent pKa is a highly influential parameter for LNP trafficking throughout the body and within the liver. Apparent pKa is dictated by the chemical identity as well as the molar contribution of each lipid component of the LNP in question. pKa has been shown to govern particle uptake by specific cell populations in the liver. Lower pKa (6–6.5), when combined with a small particle size, predominantly favors hepatocyte accumulation as the particles carry a predominantly neutral charge in the vicinity of Kupffer cells. Whereas LNPs with higher pKa (7–7.15) show preferential uptake by LSECs. Elevating the pKa above 7.3 leads to a shift in particle accumulation toward Kupffer cells as the particles become increasingly cationic.^62^, ^207^ A detailed study by Dilliard et al. investigated the impact of pKa on organ‐specific delivery of LNPs and demonstrated that the underlying mechanisms could overlap passive and endogenous targeting modalities (Figure 4).^207^ Their study showed that LNPs with apparent pKa greater than 9.2 favored lung accumulation. As the pKa dropped below 9, LNPs started shifting toward the spleen, with maximum splenic targeting achieved by LNPs with apparent pKa below 6. LNPs with apparent pKa between 6 and 7 showed preferential liver deposition. Changes in pKa change the particle charge at physiological pH (Figure 4a), which also changes the biomolecular corona composition (Figure 4b–d). This ultimately affects the main organ of accumulation for the LNPs.

Particle deformability can impact its extravasation through the endothelium. Abundant data have explored liposome deformability by incorporating edge activators to promote particle elasticity and deformability, which are described in this previous review.^208^ Yet, limited data exist on the effects of deformability in LNPs on their endothelial extravasation. LNPs formulated using an unsaturated helper lipid (DOPE) exhibit higher membrane fluidity and result in more deformable LNPs, which showed higher liver accumulation in regions with smaller endothelial fenestrations. LNPs containing the saturated lipid 1,2‐distearoyl‐sn‐glycero‐3‐phosphocholine (DSPC) had more rigid membranes and showed a preference for the spleen, which has larger endothelial fenestrations.^209^

#### 3.2.2 Biodistribution and endogenous targeting of LNPs

**FIGURE 5:**
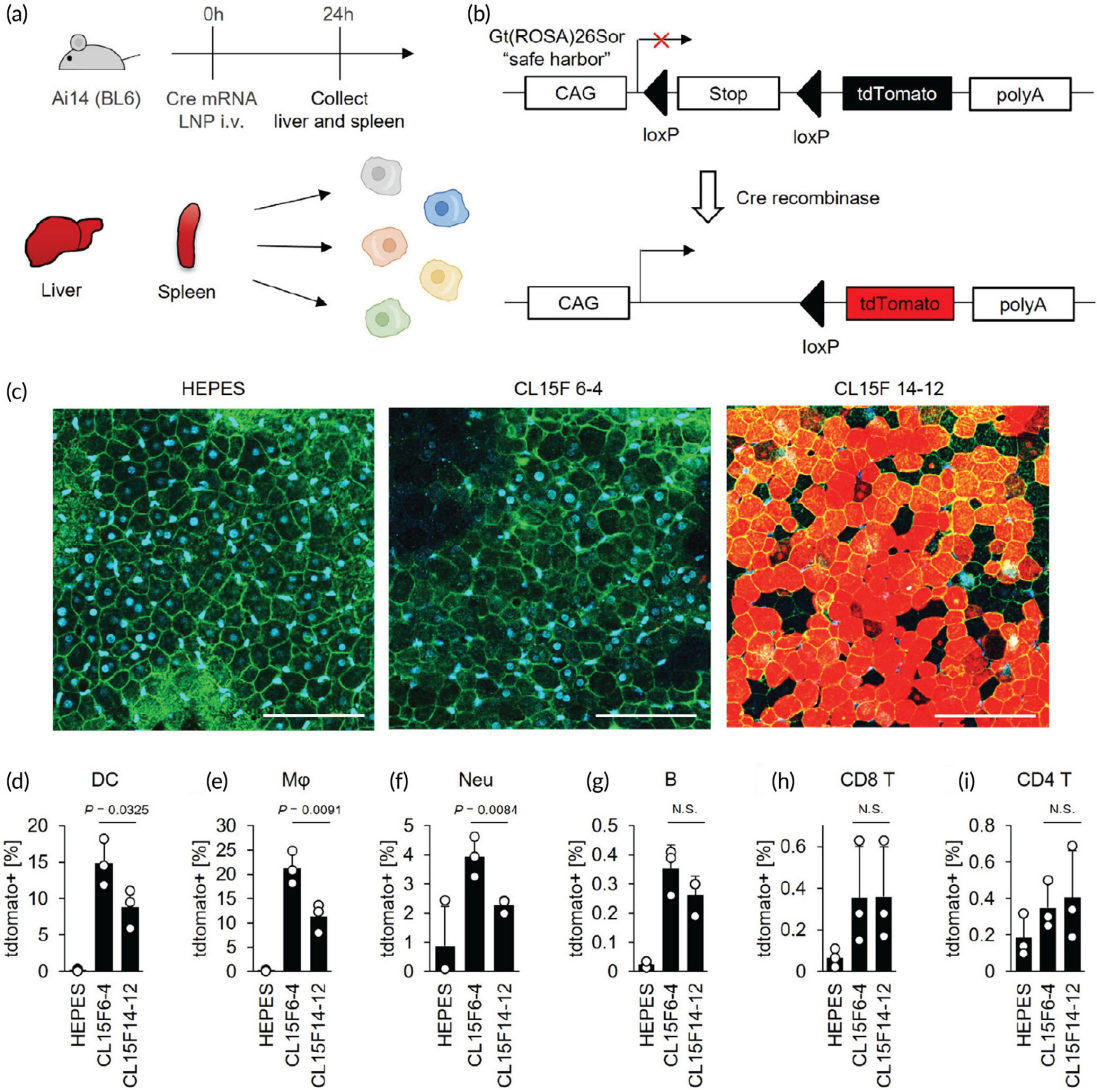
Impact of ionizable lipid tail length on endogenous targeting of lipid nanoparticles (LNPs). (a) Schematic of experimental workflow and (b) the Ai4 transgenic mouse model used for evaluating tissue tropism of LNPs with varied ionizable lipid tail length via Cre mRNA‐mediated tdTomato expression. (c) tdTomato expression in liver tissue from untreated mice (HEPES) and mice treated with 0.5 mg/kg Cre mRNA/LNPs prepared with ionizable lipids having a short tail (CL15F 6–4) or long tail (CL15F 14–12). The nomenclature “CL15F *m*−*n*” designate the overall lipid tail length (*m*) and side chain length (*n*) for each ionizable lipid. Scale bars = 100 μm. (d–i) Spleens of mice were collected 24 h after systemic administration and tdTomato expression in splenocytes were analyzed by flow cytometry. The bar graphs show the percentage of tdTomato‐positive cells in (d) dendritic cells, (e) macrophages, (f) neutrophils, (g) B‐cells, (h) CD8+ T‐cells, and (i) CD4+ T‐cells. This work was previously published by the American Chemical Society and reproduced under the terms of the CC BY‐NC‐ND 4.0 open access license.

For LNP, passive and endogenous targeting are not mutually exclusive targeted delivery mechanisms as some changes to LNP composition can change its physical properties, such as size and charge, while simultaneously transforming the biomolecular corona composition of these particles. For example, shortening the ionizable lipid tail redirected LNPs from the liver to the spleen (Figure 5a–c) via uptake in splenic dendritic cells, macrophages, and neutrophils (Figure 5d–i), regardless of the head group composition.^210^ A potential explanation for this observation is that shorter tail groups led to the formation of larger LNPs, reducing the specific surface area of the system and exposing more of its helper lipid component (DSPC) on its surface. DSPC has been reported to less readily interact with ApoE than its unsaturated analogs. Both the increased LNP size and reduced ApoE content in its biomolecular corona lead to LNPs' targeting toward the spleen rather than the liver. ApoE binding to the surface of LNPs has been shown to drive ionizable lipid components to the core and push cholesterol to the surface of the LNP.^211^ Alternatively, the binding of vitronectin or fibrinogen to the NP surface has been shown to reveal cryptic binding sites on these proteins.^212^ Receptor‐mediated uptake is coordinated by the cell membrane receptors for the components in the LNP's biomolecular corona.

High throughput screening methods have enabled investigators to understand the impact of structural variations of LNP on differential organ/tissue/cell targeting and transfection efficiency at target sites. To that end, the DNA barcoding system developed by James Dahlman and colleagues has proven an invaluable tool to broaden the design space in LNP based therapeutics.^213^ The system allows the administration of broad arrays of LNP to a single animal and the subsequent generation of transfection data of every single particle within the array on a cellular level by co‐loading a unique DNA barcode to each particle along with the reporter mRNA cargo. The unique DNA barcode can later be sequenced in collected tissue to locate the preferred target cell of each LNP design in the array and how that LNP performed in terms of transfection or gene silencing using reverse transcription quantitative polymerase chain reaction (RT‐qPCR). This method can significantly reduce the number of animals required per study and avoids complications with in vitro–in vivo correlation. Yet, some concerns regarding the potential for simultaneously administered LNP within the array to impact each other's in vivo behavior have been raised; more data is still required to validate or invalidate such concerns.

The incorporation of PEGylated lipids, as well as PEG desorption kinetics play a central role in the endogenous targeting process of LNPs. The initial presence of PEG on LNPs upon entry into the bloodstream is crucial to minimize the adsorption of opsonins, which when found in high percentages in the biomolecular corona, can lead to rapid clearance by the MPS system. Although subsequent desorption of PEG is essential to permit the subsequent steps of endogenous targeting and allow efficient particle uptake and endosomal escape, slowly desorbing PEG can also prolong the circulation time of the LNP, leading to an enhancement in its extrahepatic accumulation. Generally, PEGylated lipids with shorter hydrophobic tails (e.g., 1,2‐dimyristoyl‐rac‐glycero‐3‐methoxypolyethylene glycol, DMG‐PEG) can desorb more rapidly than their counterparts with longer hydrophobic tails (e.g., 1,2‐distearoyl‐sn‐glycero‐3‐N‐amino(polyethylene glycol), DSPE‐PEG).^214^ It should be noted that as the molar contribution of PEGylated lipids increases, the impact of tail length on the desorption rate increases.^215^ It has also been demonstrated that fast‐desorbing PEG with short tails generates less anti‐PEG IgM in mice compared to slower‐desorbing PEG with longer acyl chains.^216^ Hence, it could be valuable to tailor the kinetics of de‐PEGylation by combining PEGylated lipids with varying acyl chain lengths on a single particle to minimize potential ABC of PEGylated LNPs. ABC is an immunogenic response that has been observed upon repeated administration of pegylated LNPs mediated by MZ B‐cells in the spleen resulting in anti‐PEG IgM generation. As previously noted, much higher doses of LNPs (1000‐fold increase in protein expression) may be required for therapeutic applications (e.g., protein replacement) relative to mRNA vaccine applications, which may dictate the incorporation of much higher doses of PEG.^217^ The ABC effect at this point may become an obstacle, and careful manipulation of PEG desorption kinetics or its full replacement with less immunogenic alternatives such as poly(carboxybetaine)s and poly(sulfobetaine)s, polysarcosines, poly(phosphoester)s, and poly(oxazoline)s may be necessary.^218^, ^219^ Another alternative may be resorting to cell‐membrane‐based coatings or functionalization with anti‐phagocytic signals like CD47.

Seminal work from the lab of Daniel Siegwart demonstrated that modifying the charge of LNPs could effectively redirect them to different organs, showcasing the adaptability of LNPs in targeted drug delivery.^202^, ^207^, ^220^ These SORT LNPs incorporate a sorting lipid outside the four key lipid components of classical LNPs, while maintaining the key lipids' molar ratios constant could redirect the LNP's natural tropism away from the liver. This effect occurs independently of the sorting lipid structure and is primarily a function of the sorting lipid's net charge and molar percentage of the SORT lipid in the overall LNP composition. Their studies showed that incorporating a permanently cationic lipid such as 1,2‐dioleoyl‐3‐trimethylammoniumpropane (DOTAP), didodecyldimethylammonium bromide (DDAB), or 1,2‐dioeoyl‐sn‐glycero‐3‐ethylphosphocholine (EPC) could shift particles from the liver to the spleen (at 10%–15% molar contribution) and eventually to the lung as well as the kidney (at a molar contribution greater than or equal to 40%). On the other hand, incorporating permanently anionic lipids (e.g., 18pA, 14pA, or 18 BMP) resulted in particle tropism exclusively to the spleen, with no detectable expression in other organs. These tropisms were maintained independent of structural variations to the linker and tail groups of the sorting lipids candidates. Meanwhile, ionizable cationic lipids, such as DODAP, and C12‐200 demonstrated a maintained tendency to direct LNPs to the liver. Moreover, Cheng et al. demonstrated this trend not only in an experimental ionizable cationic lipid, but also in the clinically established Dlin‐DMa‐MC3, a two‐tailed single head ionizable lipid.^202^ Dlin‐DMa‐MC3 has been previously reported to deliver siRNA and mRNA exclusively to the liver upon IV administration, but addition of an ionizable cationic lipid altered this tropism. In terms of toxicity, all SORT LNPs showed excellent liver and kidney tolerability, as well as no rise in inflammatory cytokines following in vivo administration.

Consistent preclinical data have been obtained in mice by several research teams that support the charge‐based targeting of LNPs to various organs. LNPs with a permanently positive charge under physiological pH following PEG desorption to target the lungs, their negatively charged counterparts can achieve spleen targeting and whereas generally neutral LNPs favored liver accumulation. The method by which LNP charge was induced, whether through introduction of a sorting lipid or manipulation of existing lipid components, did not appear to affect these tropisms. For example, an alternative and more simplistic approach to adding a sorting lipid to the four original lipid components of an LNP was proposed by LoPresti et al.^221^ Their study demonstrated that replacing the helper lipid component with either a cationic, anionic or neutral helper lipid led to a significant redirection of LNPs to the lungs, spleen, and liver, respectively, regardless of the ionizable lipidoid's chemical identity. Replacing the neutral DOPE with cationic DOTAP shifted liver to lung reporter protein expression from 36:1 to 1:56, while replacing DOPE with the anionic phosphatidylserine shifted liver to spleen reporter protein expression from 8:1 to 1:3. The underlying mechanism of this organ targeting mechanism could be related to alterations in the composition of the biomolecular corona as well as the potential that some LNP components could alter immune cell trafficking to specific organs. Using a barcoding high throughput in vivo screening approach, lymph node targeting LNPs have also been identified where lead formulations possessed neutral helper lipid (e.g., DOPE) at an optimal molar ratio and generally lacked cationic lipid species.^222^ Cationic lipids, such as DOTAP, have been shown to alter the composition of the biomolecular corona of LNPs to be predominated by vitronectin. Vitronectin is a glycoprotein involved in cell adhesion and coagulation that can bind to αVβ3 integrin receptors highly expressed in the lungs.^223^ Vitronectin tends to bind the glycosaminoglycan heparin, which is abundant in serum.^224^ It is worth noting that such cationic LNP‐polyanion (heparin) interaction could lead to formation of LNP aggregates small enough to be entrapped in the narrow capillaries of the first organ it encounters (e.g., lung) following injection.

The hypothesis regarding immune cell trafficking is more related to the proposed mechanism of action of anionic lipids. Anionic lipids have been shown to enhance the immune cell infiltration into the spleen by LoPresti et al.,^221^ and further work from Alvarez Benedicto et al. who showcased spleen‐targeted SORT LNP by incorporating anionic 18:1 phosphatidic acid (PA) as a sorting lipid in their LNP.^225^ These LNPs could produce Chimeric Antigen Receptor (CAR) T‐cells in vivo in wild type mice by delivering CAR encoding mRNA to splenic CD3+, CD8+, and CD4+ T‐cells with high specificity. The same was also shown when exchanging cholesterol with an anionic analog, the naturally occurring bile acid, resulting in improved splenic tropism following IV or intraperitoneal injection.^226^

Beyond the SORT LNP approach, extrahepatic delivery can be enhanced by prolonging LNP circulation time. A relevant approach was proposed by Chander et al. who reformulated LNPs to contain a molar ratio of 40% helper lipids rather than 10%.^227^ Using either DSPC or egg sphingomyelin (ESM) as the helper lipid in such a high molar concentration altered the morphology of the LNP to feature a small solid core surrounded by a lipid bilayer. This altered conformation led to better RNA shielding in the core, higher serum stability of the particle, and prolonged circulation. Results also showed LNPs with 40% ESM significantly outperformed the Onpattro formulation in in vitro transfection (10‐fold enhancement in luminescence) as well as splenic (three‐fold increase) and bone marrow (two‐fold increase) accumulation in vivo. Isaac et al. identified an endogenous targeting strategy with vitamin D3 containing LNPs which enables targeting of the vitamin D receptor leading to highly selective delivery to the pancreas.^228^ More recently, Su et al. demonstrated that, using their own library of a degradable ester‐core ionizable lipid, they could achieve more exclusive lung targeting when they also added a permanently cationic lipid and entirely removed cholesterol from their formulation. They showed similar exclusive targeting of the liver upon re‐incorporation of cholesterol and a phospholipid into the formulation.^229^

With these established and newly discovered principles of endogenous targeting for LNPs, engineering LNPs that are enriched with endogenous proteins known to shift their biodistribution to desired organs has also been explored. To accomplish this, LNPs can be engineered to preferentially accumulate specific species such as ApoE and vitronectin to influence their fate in vivo by varying their net charge and/or lipid content as described above. For example, LNPs can be designed as a function of ionizable lipid chemistry to increase levels of ApoE in the protein corona leading to enhanced delivery to hepatocytes via LDL receptors.^207^, ^230^, ^231^, ^232^ Similarly, LNPs have also been designed in an ionizable lipid‐dependent manner which acquire a protein corona enriched in vitronectin to enable targeting the lung, kidneys, and the tumor microenvironment via uptake in integrin overexpressing cancer cells.^233^, ^234^, ^235^ In recent work, albumin‐binding LNPs were generated by substituting PEGylated lipids with an Evans blue‐modified lipid to stabilize the formulation.^236^ This LNP has shown promise for mRNA vaccine applications by minimizing hepatic accumulation while promoting albumin‐mediated lymphatic transport and receptor‐mediated uptake in dendritic cells. Another related biophysical approach is altering membrane fluidity through the incorporation of more unsaturated lipid components into the LNP. This can alter or enhance the protein corona binding to the LNP's surface. Zhang et al. demonstrated membrane fluidity alterations by exchanging the helper lipid DSPC with its unsaturated analog DOPE, which led to enhanced ApoE binding on DOPE‐containing LNPs and enhanced liver rather than spleen targeting.^209^

Lipid NP components have also been leveraged to address the inherent limitations of calcium phosphate (CaP) gene delivery technologies. CaP is an inorganic material that has been used for several decades to condense and deliver RNA as well as facilitate viral transduction, yet its physical instability limits its in vivo application. Hence, several approaches have been explored to enhance CaP's efficacy, such as coating it with polymers or lipids.^237^ Lipid coating could address the inherent instability of CaP‐siRNA complexes.^237^ For instance, a CaP‐siRNA core coated with cationic lipids, then functionalized with PEG and anisamide, resulted in a nanocarrier that effectively targeted sigma receptors in B16F10 melanoma cells. Following a single IV injection, antiluciferase siRNA led to a 78% reduction in luciferase expression in metastasized B16F10 in vivo.^238^ Another approach relied on multilayer lipid deposition on CaP cores, with an inner layer of the anionic lipid DOPA followed by a cationic or neutral lipid.^239^ This setting significantly improved the gene silencing effect and allowed the co‐loading of a chemotherapeutic agent. The lipids used in these studies are similar in composition to those used for classical LNP preparation.

### 3.3 Polymer nanoparticles

Polymer chemistry can be finely tuned to produce polymeric nanoparticles (PNP) with a desired activity and biodistribution in vivo. PNP can be derived from both natural and synthetic polymers.^240^ Commonly used natural polymers include chitosan, alginate, and dextran, while common synthetic polymers for NP formation include poly lactic‐co‐glycolic acid (PLGA), polyacrylates, and poly(ethylenimine) (PEI). However, certain polymers are better suited to gene therapy, based on properties such as those possessing cationic charge for nucleic acid compaction and cargo protection.^241^ In this section of the review, we will primarily focus on the polymers that are most utilized for delivery of genetic therapies: PEI, PLGA, poly(β‐aminoester) (PBAE), poly‐l‐lysine (PLL), and chitosan. While various PNPs have shown great promise in preclinical testing, there is currently a translational gap regarding the approval of these gene carriers for use in humans. However, polymer NPs may have a manufacturing advantage over AAV and LNPs. In addition, PNPs have been more widely tested and proven effective for delivery of DNA‐based cargoes for gene therapy applications.

#### 3.3.1 Synthetic PNPs

While many new non‐viral gene delivery approaches have emerged in the past approximately three decades following its discovery, PEI is one of the most robustly studied systems for polymer‐mediated encapsulation of nucleic acids for gene delivery.^242^ PEI is a cationic polymer consisting of monomers of ethylenimine which are covalently linked together to form the PEI backbone and can be arranged in linear or branched architecture with varying overall polymer MW. PEI polymers with MW of 20–25 kDa in linear or branched form have been shown to be highly efficient at transfection of mammalian cells.^242^ Previous work has shown that the cationic PEI is able to effectively and spontaneously complex with negatively charged DNA or other nucleic acids (e.g., siRNA and mRNA). In more recent work, Binder et al. utilized molecular dynamics simulations to determine the thermodynamics of PEI and siRNA complexation.^243^ They simulated complexes with PEI of varying MWs and degrees of branching where they validated that the complexation of PEI and siRNA is thermodynamically favorable. In addition, they showed that the reaction was even more favorable at pH similar to endolysosomal conditions than at neutral pH. However, significant concerns remain on the associated toxicity and proinflammatory responses associated with PEI‐based PNP. To address this, lower MW (e.g., 2 kDa) PEI can be crosslinked using bioreducible linkers (e.g., disulfide‐linked) to achieve optimal DNA/RNA packaging efficiency while reducing the cytotoxicity of the resulting PNP.^244^, ^245^

PBAEs are a unique class of biodegradable polymers that have been developed for gene delivery applications. PBAE structure is an amine‐containing polyester, with a highly modifiable structure.^246^ Lynn and Langer detailed their rationale for the synthesis of PBAEs, basing it upon a poly(ester amine) approach by combining esters and amines via Michael Addition reaction enabling production of a diverse library of polymers.^247^ Importantly, PBAEs are biodegradable which addresses one of the major drawbacks of conventional, nondegradable PEI‐based PNPs. In regards to their performance, PBAE is highly tunable with different amine/acrylate combinations producing PNP with unique DNA compaction and endosomal escape capabilities. Moreover, PBAE gene delivery efficiency and targeting to specific tissues can be modified by altering the terminal end‐capping groups which is highly advantageous for application‐specific design and optimization. We will further elaborate on specific design modifications for organ‐selective targeting of PBAE PNP in Section 3.3.3. In initial work to establish the clinical relevance of this system, Green et al. illustrated an optimized PBAE PNP‐mediated gene delivery comparable to adenoviral gene vectors in vitro and it has gone on to be successful in a wide range of preclinical in vivo models.^248^

PLGA is a biocompatible and biodegradable polymer used to fabricate PNP for both drug and gene delivery. PLGA is a block copolymer consisting of lactic and glycolic acid subunits, which allows for a uniquely tailorable polymer for NP formulation. The polymer MW, monomer ratios, and monomer MWs can all be altered through polymer synthesis. Cun et al. described a method of encapsulating siRNA into PLGA NPs using double‐emulsification.^249^ They achieved >50% encapsulation efficiency and a particle diameter of 250 nm, demonstrating that PLGA NPs provide an avenue for delivery of siRNA and other nucleic acid cargoes. One advantage of PLGA over other PNP is that PLGA can be readily used for combined delivery of small molecules and nucleic acids. Heo and Lim showed that PLGA NPs could be used for simultaneous delivery of siRNA and imiquimod for the immunomodulation of dendritic cells.^250^ This potent combination therapy outperformed the commercial control in silencing the immunosuppressive STAT3 gene.

Another biodegradable polymer system receiving considerable attention for nucleic acid delivery is poly(amine‐co‐ester) (PACE). Zhu et al. were able to establish relationships between the MW of the PACE polymer and its ability for effective complexation with mRNA.^139^ In addition, they showed that PACE polymers with a MW of 5–10 kDa were most effective at promoting endosomal escape for efficient mRNA delivery. Piotrowski‐Daspit et al. screened a library of PACE polymers with varied MW and functionalization to generate PNPs to evaluate their blood concentration over time and tissue biodistribution within mice after systemic administration.^251^ Overall, they noted that it was very difficult to reduce liver and spleen accumulation of PACE PNPs, regardless of circulation time changes. However, an increase in retention time in the blood may allow for additional PNPs to reach more difficult‐to‐reach sites such as the bone marrow, heart, or lungs. Overall, they determined that the polymer chemistry and NP characteristics strongly impacted the fate of PNPs and delivery ability of nucleic acid therapeutics due to their impact on retention time and tissue distribution.

An emerging body of work focuses on zwitterionic PNPs. Zwitterions feature positively and negatively charged regions within their structures yet are overall neutral in charge.^252^ This property can allow them to facilitate nucleic acid encapsulation efficiency rivaling cationic polymer yet at much lower cytotoxicity. Shen et al. developed a zwitterionic polymer‐based polymeric micelle.^253^ This system switched surface charge in acidic environments such as the endosome. When delivering plasmid DNA, the zwitterionic polymer micelles showed effective gene transfection and low cytotoxicity in acidic pH. Zwitterionic modifications can also be made to existing polymers used for nucleic acid delivery. For instance, Liu et al. modified PEI with zwitterionic molecules in an attempt to reduce the cytotoxicity associated with PEI.^254^ These NPs showed a significant reduction in cytotoxicity when tested in vitro, although with slightly less effective gene silencing for encapsulated siRNA cargo. Zwitterionic polymers could yield a promising platform for gene therapies, especially for navigating acidic environments such as the tumor microenvironment and the endo/lysosome.

Similarly to zwitterions, charge‐altering releasable transporters (CARTs) are an emerging class of polymers used for nucleic acid delivery due to their ability to change their charge depending on their environment. McKinlay et al. reported on the use of CARTs to deliver mRNA, in which the polymeric material initially conveys a positive charge in order to complex with the negatively charged mRNA.^255^ Upon delivery into the cell, the CARTs undergo a charge‐altering mechanism that converts the positively charged PNPs into neutral small molecules, releasing the mRNA contained within NP. It is believed that the CARTs enable more efficient endosomal escape, owing to their changing charge. In addition, CARTs have been shown to exhibit organ‐specific targeting of nucleic acids with specific modifications to their polymer backbone.^256^ Several past studies have reported targeted delivery to the lungs or spleen, depending on the CART properties. While glycine‐based CARTs show enhanced retention and transfection in the spleen when delivered systemically, lysine‐based CARTs exhibit higher expression levels in the lungs.^255^, ^257^ Other alterations, such as guanidinylated serinol CARTs, showed selectivity for either the spleen or the lungs, depending on specific properties of the CART, such as block length, charge ratio, and degree of polymerization.^256^

CaP‐based gene vectors, previously discussed in the context of LNPs, have also shown promise when used in combination with PNP‐based therapeutic approaches. For example, Giger et al. explored the incorporation of PEG‐conjugated alendronate to coat CaP‐siRNA NPs and could demonstrate that, despite being taken up predominantly via clathrin‐mediated pathways, these particles could still exert potent gene silencing in vitro.^258^ These CaP‐siRNA NPs achieved ~80% VEGF mRNA knockdown in vitro in pancreatic cancer cells (PanC‐1). Pitella et al. demonstrated that a CaP‐siRNA system co‐assembled with a pegylated charge‐conversional polymer (CCP) showed high siRNA loading and rapid endosomal escape.^259^ These particles enabled significant VEGF silencing (68% knockdown) in subcutaneous BxPC3 tumors following systemic administration in vivo.^260^ PEI modification is another approach to stabilize CaP‐based RNA delivery systems further and enhance their transfection performance. This was demonstrated by Devarasu et al., who reported the fabrication of a CaP‐based system coated with alternating layers of siRNA and PEI.^261^ This system showed potent 95% luciferase silencing in vitro and in a murine tumor xenograft model in vivo.

#### 3.3.2 Natural PNPs

While synthetic polymers have remained popular for gene delivery applications given their ability to be manipulated in a controlled and systematic fashion, many naturally derived polymers have garnered attention for use in PNP formulation. PLL is a cationic polypeptide that has been widely studied for gene delivery purposes.^262^ Due to the positively charged nature of PLL, it easily complexes with DNA and RNA to form NPs that can be delivered intravenously.^263^ It has been determined in prior work that PLL containing >20 amino residues in length can efficiently condense plasmid DNA in PNP with diameters below 100 nm at physiological ionic strength and pH. To improve the stability and biocompatibility of PLL‐based PNP, PLL‐PEG copolymers are often used to reduce their net surface charge and prevent their aggregation. One of the most clinically advanced PNP consists of a 30‐mer PLL with an N‐terminal cysteine conjugated to a 10 kDa PEG (CK30) which has been tested in the context of inhaled gene delivery^264^ and gene delivery to the CNS.^265^

Chitosan is a naturally occurring polysaccharide that can be found in crustaceans, fungi, and insects.^266^ Chitosan remains a popular natural polymer for the encapsulation and delivery of gene therapies. An early study by Erbacher et al. demonstrated that chitosan could condense and complex DNA into NPs in the range of 50–100 nm, proving chitosan's promise for gene delivery.^267^ These nano‐complexes effectively transfected HeLa cells, maintaining transgene expression for 96 h. Furthermore, Rudzinski et al. evaluated chitosan NPs for their ability to deliver siRNA to HCT‐116 colon cancer cells.^268^ They showed that chitosan PNP had comparable transfection efficiency to Lipofectamine 2000 and were able to effectively reduce the expression of β‐catenin, a protein associated with colon cancer spread. In a similar comparison, Redhwan et al. utilized chitosan to encapsulate siRNA with the ultimate goal of reducing apoptosis caused by hyperglycemia.^269^ The siRNA encapsulated within chitosan PNP exhibited a higher transfection efficiency than the naked siRNA in Neuro2A cells. When compared to lipofectamine, the chitosan NPs were able to achieve comparable knockdown of target genes and reduced apoptotic markers levels observed in the untreated control group. Zhou et al. prepared chitosan NPs with double stranded DNA (dsDNA) cargo.^270^ In their work, they determined that the dsDNA‐containing chitosan NPs activated clathrin‐mediated endocytosis through the upregulation of the clathrin heavy chain gene. This study was the first of its kind to determine the mechanism by which dsDNA is delivered into cells.

Other polypeptide systems have also shown promise as carriers with several advancing toward clinical impact. Peptide‐mediated gene delivery can be sub‐classified into cationic peptides, amphipathic peptides, RNA covalent conjugates, and phase‐separation systems. The most prominent cationic peptide in clinical trials is protamine, which has been evaluated for mRNA delivery in non‐small cell lung cancer (NCT00923312 and NCT01915524), metastatic melanoma (T00204607), prostate cancer (EudraCT 2008‐003967‐37 and NCT0187738), and rabies (NCT02241135).^271^, ^272^ Synthetic cationic peptides that tried to improve the overall performance featured branching and histidine residues (e.g., H3K(+H)4b), which improved particle assembly as well as endosomal escape simultaneously.^273^ Overall, many of these cationic peptides exhibit lower transfection performance than other systems, potentially due to limited cytosolic mRNA release.^274^ In contrast to predominantly cationic peptides, amphipathic peptides feature both cationic and hydrophobic regions, with the hydrophobic regions playing a stronger fusogenic role at the plasma and endosomal membranes through hydrophobic interactions.^275^, ^276^, ^277^

Sun et al. reported the development of a conjugate peptide based on the pH‐responsive, self‐coacervating, histidine‐rich HBpep peptide, modified by lysine insertion at position 16, shifting its isoelectric point from 7.5 to 9, thereby rendering the system monomeric at physiological pH.^278^ They then further conjugated the lysine head with a disulfide bond containing a hydrophobic group that induces self‐coacervation of the system at near neutral pH, where it aggregates by liquid–liquid phase separation to form micrometer‐sized coacervates at physiologic pH. Yet the disulfide modification renders the system self‐immolative under the reducing glutathione‐rich intracellular environment. Their system could affect energy‐independent endocytosis of peptide‐mRNA coacervates, followed by glutathione‐mediated release directly into the cytosol. Miliotou et al. presented direct conjugation of in vitro transcribed RNA to protein transduction domain (PTD, peptide sequence PFVYLI).^279^ They argued that the covalent conjugation rather than mere electrostatic assembly approach of the peptide carrier and the RNA cargo, although more chemically limiting, could lead to reduced cargo loss to RNase degradation. Their system showed promise in an in vitro model of β‐thalassemia.

#### 3.3.3 Structure and function of PNP

Cationic polymers are largely associated with effective gene delivery. El‐Kharrag et al. showed that there was a positive correlation between NP charge and transfection efficiency. All NPs tested were positively charged, and as the magnitude of the positive charge increased, the percent of transfected human hematopoietic stem and progenitor cells increased.^280^ However, one of the main issues with cationic polymers is cytotoxicity.^281^ While this is observed with cationic PNP regardless of their therapeutic cargo, this can prove especially problematic for PNP‐based gene therapies. A large body of research has looked at the cytotoxicity of PEI specifically in the context of gene delivery. This is due to the positively charged polymer associating with and compromising the negatively charged cell membrane.^282^ At neutral pH, PLGA does not have the positive charge required to complex the anionic RNA phosphodiester backbone. Thus, to utilize PLGA as an RNA delivery system, scientists have added cationic chemical groups such as chitosan to deliver siRNA in mice.^4^

There are conflicting reports on the impact of NP size on gene delivery abilities. Ogris et al. generated DNA/transferrin‐PEI complexes of varying sizes, from 40 to >1000 nm.^283^ Using these complexes to reduce luciferase gene expression, the small particles showed at least a 10‐fold reduction in multiple cell lines. This suggests that smaller NPs have better transfection abilities than large NPs. In a separate study, Ogris et al. studied DNA/PEI particle size and found conflicting results.^284^ They showed that large, aggregated complexes showed higher levels of gene expression. As such, the impact of NP size on transfection efficiency will be largely context dependent, relying on polymer material and target cell type. Moreover, the differences in cargo loading for larger versus smaller polyplexes make direct comparisons of transduction efficiency in specific cell types challenging.

The MW of the polymers used in NPs has been shown to impact cytotoxicity and delivery. When looking at gene delivery specifically, the differences due to MW can greatly affect transfection abilities. For instance, Choi et al. investigated chitosan and RNA polyplexes with varying MWs of chitosan as an immunotherapy for cancer. They found that the highest MW chitosan had the greatest effect, resulting in the largest immune response among the polyplexes tested.^285^ While a large immune response can be beneficial for immunomodulatory therapeutics, a dramatic increase in immune cell infiltration can prove harmful in other situations. In fact, while high MW polymers are associated with higher transfection efficiency, they can also lead to high cytotoxicity and poor nucleic acid cargo release at the target site.^286^ Breunig et al. found that incorporating disulfide bonds within the polymer structure resulted in lower toxicity.^287^ In the reductive environment within the cell, the high MW polymer NPs were broken down to lower MW polymers which are less cytotoxic and better tolerated. Alameh et al. found that an increase in the MW of chitosan resulted in higher NP internalization within cells and more knockdown by the siRNA cargo.^288^

Similar to LNPs, pKa, or acid dissociation constant, significantly impacts the ability of PNPs to deliver nucleic acid payloads.^289^ During endosomal maturation, the pH of the endosome becomes extremely low. This presents a threat to nucleic acid stability, and the pKa of the polymer allows for buffering of the endosomal pH to a range that is more suitable for nucleic acids. A study by Du et al. studied a range of triblock copolymers with pKas ranging from 5.2 to 7.0. This study found that polymers with intermediate pKa values of 5.8–6.2 provide the best gene silencing via siRNA delivery and validated that this was due to more efficient endosomal escape.^290^ However, the exact pKa range that enables the most efficient endosomal escape is likely to depend on the carrier, as well as the tissue in which the nucleic acid is being delivered.^291^ In addition to pKa, other polymer properties have been altered to increase endosomal escape. Chiper et al. modified low MW PEI polymers to create polyplexes that dissolved at low endosomal pH, while buffering the pH within the endosome to protect nucleic acids from degradation.^292^ They went on to use this PEI PNP platform as a treatment to deliver nucleic acids to the muscle to treat Duchenne's muscular dystrophy in a mouse model.

One popular method for improving the efficiency of polymer nanocarriers is to increase the branching of the polymer. This is achieved by creating more end groups to the polymer, as opposed to the traditional linear polymer with only two end groups. Much work has been done to investigate the effects of branching of PEI and PBAE, with this structural modification also being studied in other polymer systems. Hyperbranched PBAE (HPBAE) has been studied at length for gene delivery in a range of cell and tissue systems. Li et al. found that removing the low MW (<15 kDa) components when formulating HPBAE PNP increased transfection efficiency.^293^ Li et al. also found that branch unit distribution, a structural property of HPBAE, plays an important role in transfection efficiency.^294^ A more uniform branch unit distribution of HPBAE resulted in a higher transfection efficiency. HPBAE PNPs have also been investigated and functionally tested as a carrier for siRNA gene silencing. Ooi et al. showed that HPBAE provides a better alternative to linear PBAE for siRNA delivery, utilizing a lower siRNA dose and lower polymer/siRNA ratio.^295^

Building upon this work, Liu et al. utilized the HPBAE polyplexes encapsulating minicircle DNA to establish feasibility of these polyplexes to deliver genetic material to the brain.^296^ The HPBAE was modified with disulfide bonds to promote rapid degradability, which did not negatively impact the cytotoxicity of the polyplexes. The minicircle DNA was delivered via polyplex to multipotent adipose‐derived stem cells and astrocytes, and high transfection efficiency was observed of >70% and >50% transfection efficiency, respectively. Rui et al. also demonstrated that HPBAEs can be modified for further optimization and expand its utility even beyond nucleic acid delivery.^297^ Utilizing a carboxylated PBAE system, they encapsulated a protein for cytosolic delivery and CRISPR/Cas9 for genetic engineering within the nucleus. They achieved over 75% gene knockout across various cell types, indicating that further modification of HPBAEs could lead to more effective protein delivery, which has proven difficult in the past. HPBAEs also show promise for large‐scale manufacturing. Zeng et al. found that lyophilized and frozen HPBAE particles still showed potent transfection abilities up to 1 year after formation.^298^

High throughput screening has proven essential in the design and testing of PNP as gene delivery vehicles. Ulkoski et al. developed a robotic‐assisted automated high throughput screening method to investigate several important characteristics of PNPs for delivery of mRNA including cytotoxicity, cellular uptake, transfection efficiency, nucleic acid encapsulation efficiency, and particle size.^299^ Through this screening, they uncovered structure–function relationships where copolymers with increasing charge density and optimal hydrophobicity by controlling alkyl side chain length led to enhancements in mRNA encapsulation efficiency. Rodrigues et al. evaluated various polymer NP formulations for effective mRNA delivery to fibroblasts.^300^ They tested 152 formulations using their high throughput screening method to determine their effectiveness for delivering a Cre recombinase mRNA in a mouse fibroblast Cre‐loxP reporter cell line. From these 152 formulations, they were able to rapidly eliminate cytotoxic compounds and compare the effectiveness of lead formulations to commercial controls (Lipofectamine) based on Cre‐mediated GFP expression. They identified a lead candidate which mediated significant mRNA delivery to fibroblasts in vitro based on its high buffering capacity to promote endosomal escape and cytosolic mRNA release. Using this lead PNP, their group went on to demonstrate significant mRNA delivery to activated skin fibroblasts following intradermal injection in tdTomato mice. Rui et al. developed a new high throughput screening assay for simultaneous determination of PBAE PNP cellular uptake and endosomal escape in vitro.^301^ This assay was highly predictive of mRNA delivery abilities of PBAE variations, and they were able to screen for top PNP formulations. Following identification of lead candidates, they found PBAE NP formulations with greater polymer backbone hydrophobicity mediated higher levels of mRNA‐mediated Cre expression as indicated by tdTomato expression in Ai9 mice following tail vein injection.

PEGylation strategies are often utilized with PNP to improve their stability in vivo and enhance circulation time (to be discussed further in Section 3.3.4). However, PEG‐coated PNP have been found to have lower cellular uptake efficiency in certain instances and these tradeoffs are important to consider when developing a PNP gene delivery system. Glodde et al. observed no difference in PEI‐PEG polyplex stability between polyplexes formed with 2 and 25 kDa PEI, but the stability of the polyplex with high PEI MW was greatly reduced with increased PEG grafting.^302^ Iqbal et al. found that PEGylating PBAE NPs did not significantly decrease their uptake in three different cell lines.^303^ PEGylated PBAE showed a higher DNA transfection capacity, as well as a lower cytotoxicity when compared to PEI. These results did not include in vivo work, which would be useful to capture the whole picture of systemic delivery of PEGylated NPs for gene delivery. Indeed, Grun et al. found that in vitro results did not translate in vivo when investigating the incorporation of PEG for PACE polyplexes.^304^ They were able to conclude that there was an optimal amount of PEG grafting to improve polyplex stability while not drastically decreasing transfection abilities, between 0.25% and 5% wt% PACE‐PEG, and the impact of PEGylation on transfection was also affected by the mode of delivery of the NPs. As determining the optimal PEG amount can vary due to a variety of factors, Dogan et al. developed an artificial neural network to model the transfection efficiency depending on PEG MW and concentration, as well as NP concentration.^305^

#### 3.3.4 Endogenous targeting and biodistribution of PNPs

**FIGURE 6:**
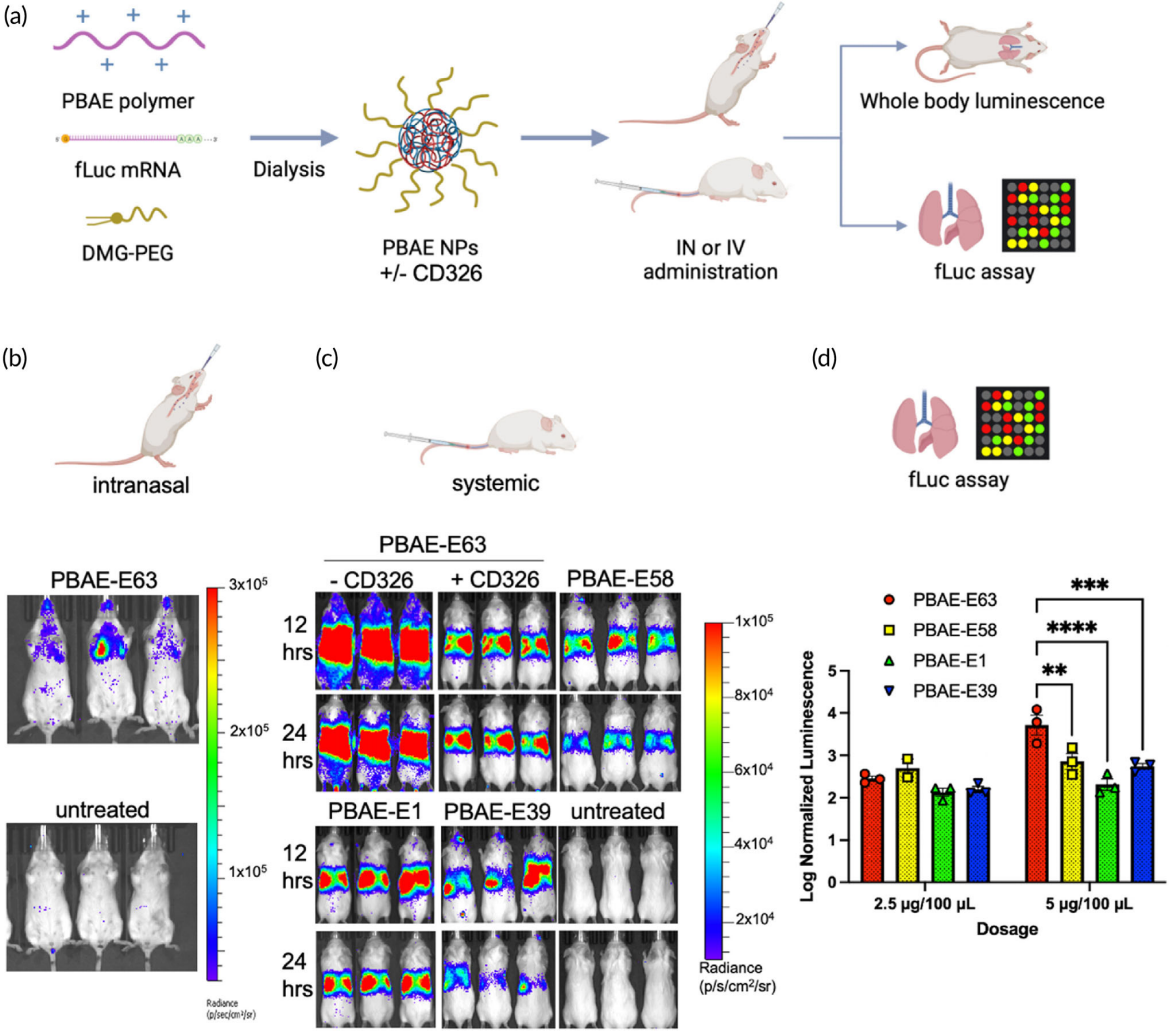
Impact of poly(β‐aminoester) (PBAE) polymer end group on lung‐specific delivery upon intravenous and intranasal administration. (a) Schematic of nanoparticle formulation and in vivo experimental workflow. (b) Intranasal administration route and resulting luciferase images with PBAE‐E68 polymer. (c) Systemic administration route via tail vein injection and resulting luciferase expression with PBAE terminated with four different end groups. +/− CD326 refers to the addition or omission of targeting ligand anti‐CD326. (d) Overall log normalized luminescence of mouse lungs after systemic administration of four different end groups and two dose concentrations. **p<0.01, ***p<0.001, and ****P<0.0001. Reproduced with permission from Elsevier. IV, intravenous; PEG, polyethylene glycol.

Polymers are easily modified, resulting in large numbers of potential PNPs for gene therapies with specific tropism to disease‐relevant organs in vivo. In particular, PBAE PNPs are notable for organ‐specific delivery to the liver, lung, brain, spleen, and other organs.^306^ While the structure is consistent across PBAE molecules, many of the other aspects of the structure can be altered such as hydrophobicity, charge density, and functional terminal end groups. Recently, Kim et al. created a library of PBAE polymers that combined different monomer variations and screened a library of PBAE PNPs for luciferase mRNA delivery efficiency following intramuscular injection in the hind limbs of mice.^307^ Through this screen, PBAE PNPs containing amphiphilic amines were identified which achieved up to 2 weeks of mRNA expression and limited off‐target delivery to the spleen or liver, significantly outperforming a conventional LNP formulation in both regards. Kaczmarek et al. utilized a library of 30 PBAEs formulated with different end‐capping groups, diacrylate:amine ratio, and alkylamine hydrocarbon length to investigate their impact on pulmonary delivery of mRNA and DNA following IV injection.^308^ Two PBAEs PNP formulations, incorporating only slight variations in the diacrylate‐amine backbone chemistry, were identified as the most efficacious for DNA and mRNA delivery in vivo illustrating how PBAE polymer chemistry dictates PNP gene delivery. Interestingly, overall luciferase expression was significantly higher for mRNA as compared to DNA independent of the specific PBAE PNP carrier utilized. Kavanaugh et al. recently determined the end‐capping of PBAE with diethylenetriamine enables PBAE PNPs to target the lung following systemic administration and with three repeated doses, achieved successful gene editing of ≥10% disease‐relevant cell types including bronchial and alveolar epithelial cells (Figure 6).^309^

In a similar effort to determine how the chemical composition and synthesis of HPBAEs impacts organ targeting, Yong et al. created a library of over 200 HPBAEs containing varying structures synthesized from different components.^310^ With this library, they efficiently screened NPs for their ability to target specific organs in vivo. They found that the most important factor in organ targeting with HPBAEs was the terminal amine structure. Based on this structure, PBAE PNPs containing ethylenediamine and morpholine terminal amines were able to target mRNA to the spleen or liver, respectively. PBAE PNPs containing methylpentane terminal amines showed a much broader activity and efficient mRNA delivery to the liver, spleen, and lungs. While the majority of investigation into polymer branching has been done with PBAE, the findings from studying HPBAE suggest that branching of other polymers used for gene delivery could be useful in increasing transfection efficiency and specific organ targeting.

Similarly to PBAE, the structure of PEI has been manipulated to investigate the impact of branching on transfection efficiency. However, branched PEI has been associated with higher levels of cytotoxicity than other branched polymer systems. This can be overcome using targeting moieties (see Section 4 for further details). Zou et al. successfully delivered DNA to the lungs using a linear PEI complex that was systemically administered via tail vein injection.^311^ These studies and many others paved the way for a large body of work utilizing PEI as a basis for gene therapy. For instance, Wong et al. furthered PEI research with their investigation of transfection efficiency in mouse neonates.^312^ Many genetic disorders are present at birth and providing gene therapies to the immature immune system of infants could reduce the immune response to these foreign materials. Similar to conventionally used adult mice, systemically delivered plasmid DNA carrying PEI complexes showed high levels of transfection in the liver in mouse pups, and transfection was also found in various other organs of interest, including the spleen, lungs, and brain, although to a lesser extent than in the liver.

**FIGURE 7:**
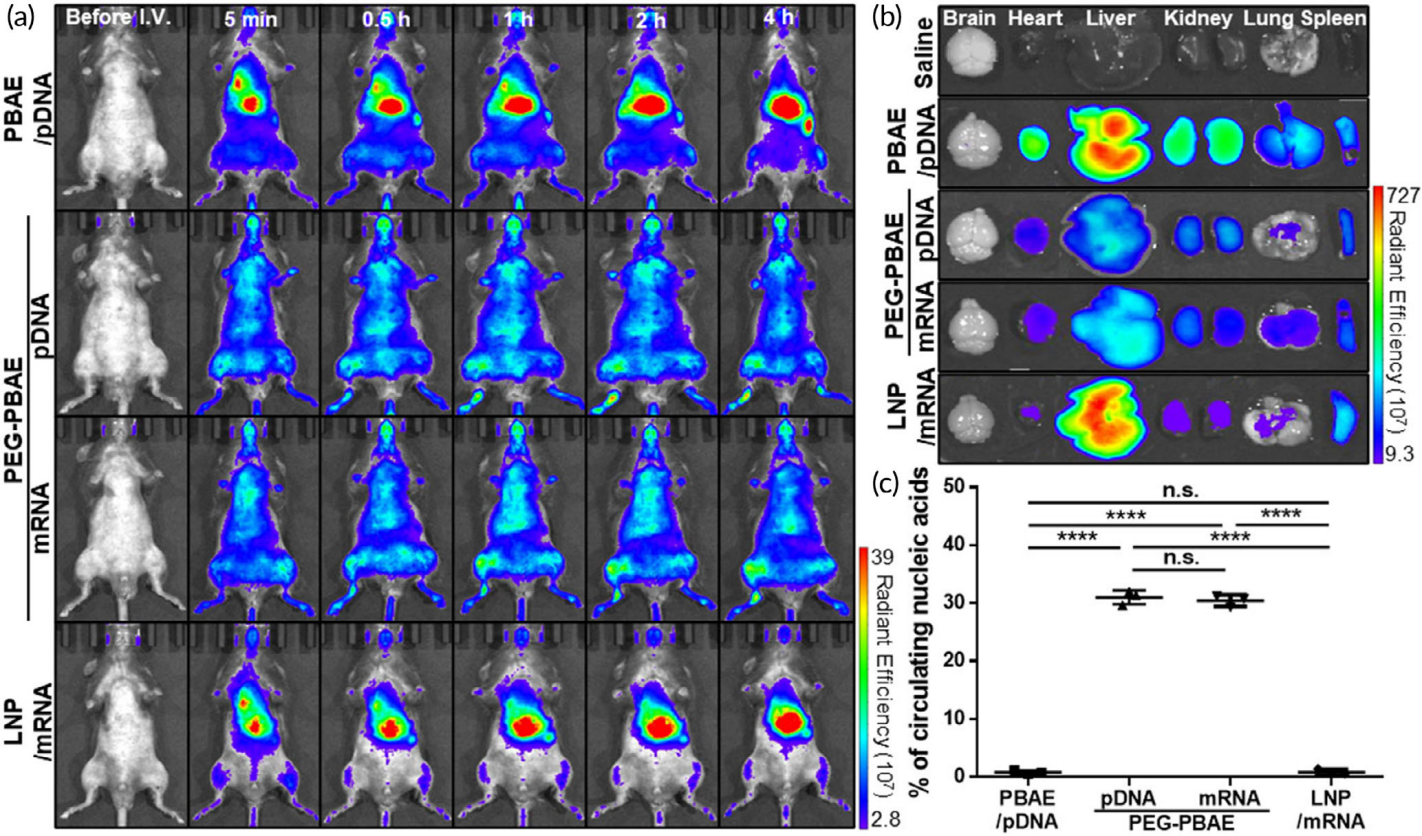
Densely PEGylated poly(β‐aminoester) (PBAE) PNP achieve significantly longer circulation time in vivo as compared to their unPEGylated counterparts and conventional lipid nanoparticles (LNPs) following intravenous (IV) administration. (a) IVIS imaging of mice over a 4 h timeframe following IV administration of unPEGylated PBAE, polyethylene glycol (PEG)‐PBAE, and LNPs carrying Cy5‐labeled pDNA or mRNA. (b) Ex vivo IVIS imaging of brain, heart, liver, kidney, and spleen following IV administration of PBAE PNP and LNPs. (c) Percentage of injected dose retained in serum after 4 h quantified via fluorescence measurements of Cy5‐labeled pDNA or mRNA. ****P<0.0001. Reproduced with permission from the American Chemical Society.

PEG‐coated PNP are not as easily taken up by immune cells, helping the NPs to circulate in the bloodstream for longer periods of time and giving a higher chance for extravasation and delivery of their cargo to target sites.^313^ In addition, the PEG chains on the NP surface reduce protein adsorption, also increasing the stealth properties of PNP. PEG can be used to neutralize the absolute zeta potential of NPs, as it is a hydrophilic polymer with net zero charge. When PEG was added to polyion complexes, there was lower cytotoxicity.^314^ Notably, the coating of PEG on NPs has been found to alter the protein corona that forms on NPs, which impacts where particles ultimately end up.^315^ A study by Perry et al. investigated how PEG grafting density impacted biodistribution of polymeric nanogels.^316^ At low density, PEG forms a mushroom conformation on NP surfaces, and at high density forms a brush conformation. Interestingly, there did not appear to be a significant difference in blood retention and biodistribution between low‐ and high‐density PEG‐grafted NPs. In general, PEGylated NPs circulated in the blood for much longer than non‐PEGylated NPs, remaining for greater than 24 h as compared to non‐PEGylated particles, which were not detected in the blood after 3 h post‐administration. The biodistribution was also altered at 24 h, with nearly all non‐PEGylated particles accumulating in the liver. While a large majority of the PEGylated particles also accumulated in the liver, NPs were also observed in the blood, spleen, lung, and kidney at higher rates than non‐PEGylated NPs. Recent work from Kwak et al. has also shown densely PEGylated PBAE PNP are retained in circulation over a significantly longer timeframe than PBAE PNP without a PEG coating as well as conventional LNPs^317^ (Figure 7). Notably, ~30% of injected dose was retained in the bloodstream after 4 h using PEGylated PBAE PNP whereas only <1% remained for unPEGylated PBAE PNPs and the conventional LNPs. A study by Mosquiera et al. found similar results in mice, with PEGylated nanocapsules (NCs) showing higher blood circulation at 24 h than poloxamine‐coated NCs. The PEGylated NCs also exhibited less liver accumulation, a common site of clearance from the blood.^318^ We note the findings in this study and others will be dependent on the NP onto which the PEG is conjugated, as well as the MW of the PEG used.

Azzam et al. found that chitosan NPs have a natural propensity for accumulating in collagen.^319^ They delivered siRNA to the liver using chitosan NPs but found that modification of the NP with a peptide increased deposition within the cell. Ward et al. evaluated the effect of PLL MW on DNA delivery in mice and rats.^320^ Specifically, 20 and 211 kDa MW PLL were complexed with three different types of DNA. They then evaluated how PLL MW and DNA type impacted delivery to various organs after systemic administration. When compared to free DNA, all complexes with the 211 kDa PLL showed increased deposition in the carcass (the body mass after main organs were removed) and decreased deposition in the liver. As previously discussed, extrahepatic delivery proves to be a challenge for gene therapies. These results indicate that PLL could be a promising avenue for gene therapeutics particularly given its past testing in clinical trials. As previously noted, Shen et al. compared cationic and zwitterionic polymers for the delivery of mRNA to the spleen and lymph nodes.^253^ When evaluated in vitro in comparison to cationic NPs, the zwitterionic polymer NPs showed a 39,500‐fold increase in protein expression and selective delivery to the spleen and lymph nodes in vivo. These findings suggest that capitalizing on the potential of zwitterionic polymers could prove highly beneficial for gene delivery.

## 4 CELL AND ORGAN‐SPECIFIC ACTIVE TARGETING STRATEGIES

Active targeting typically relies on surface coating of gene vectors with targeting entities such as peptides, antibodies or antibody fragments, aptamers, and polysaccharides, which can deliver these platforms to specific cells via uptake through receptor‐mediated endocytosis to enhance their accumulation and retention at target sites. We note a number of these approaches, in particular for antibody or peptide‐based targeting, are amenable to AAV, LNP, and PNPs.

### 4.1 Antibody‐mediated targeting

Full‐length IgG is commonly used for active targeting due to its long half‐life and improved tissue penetration, aided by receptor‐mediated recycling in endothelial cells. Active targeting with AAV using full‐length IgG antibodies has been attempted to mediate targeting to specific cell surface receptors. Ried et al. employed the IgG binding domain A and inserted the protein into the AAV2 capsid, resulting in a modular AAV‐based platform that could be coupled to IgG antibodies targeting a variety of cell types.^321^ Using this strategy, AAV2 bearing antibodies targeting CD29, CD117, and CXCR4 achieved targeting specificity and higher transduction efficiency in several hematopoietic cell lines which are not naturally permissive to wildtype AAV2. Kuklik et al. also employed the use of an epitope derived from human proprotein convertase subtilisin/kexin type 9 (PCSK9) and inserted it into the capsid protein loops of AAV vectors to create an antibody‐mediated targeting effect.^322^ They employed this system to target fibroblast activation proteins which is a relevant in many forms of cancer and found that the system led to the successful selectively targeting of AAV to transduce receptor positive cells.

Many past efforts have focused on engineering LNP–antibody conjugates to achieve precise active targeting. Breda et al. prepared IgG‐grafted LNPs as a targeting ligand toward the CD117 receptor displayed on the surface of HSCs.^323^ This system could achieve in vivo gene targeting with approximately three‐fold improvement in HSC targeting accuracy compared to an isotype antibody decorated LNP, achieving almost full hematopoietic sickle cell correction using a Cre recombinase mRNA reporter system. There were also recent efforts by Rurik et al. to engineer CAR T‐cells in vivo by delivering CAR encoding mRNA specifically to T‐cells using CD5 IgG‐grafted LNPs.^324^ These LNPs led to in vivo generation of transient CAR T‐cells that can specifically recognize and eliminate fibrotic cells in the cardiac tissue, fully restoring cardiac function in a cardiac fibrosis mouse model. Tombácz et al. demonstrated a 30‐fold increase in CD4+ T‐cell transfection upon targeting with CD4 conjugated LNPs, an approach that can provide immense benefit in modulating CD4+ cells for immunotherapy of tumors, infectious diseases, and immune disorders.^325^ In a recent study by Zamora et al., endogenously lung targeted DOTAP containing LNPs, actively lung targeted aPECAM functionalized LNPs and an LNP featuring both DOTAP and aPECAM functionalization were prepared and compared for lung targeting efficiency.^326^ They could demonstrate that either targeting mechanism when used individually led to almost equal lung accumulation as well as luciferase expression levels, with the actively targeting LNPs showing higher affinity to endothelial cells. Yet, combining both targeting mechanisms on a single particle doubled their lung accumulation compared to single targeting approaches. This approach also ameliorates first‐pass organ accumulation of endogenously targeted LNPs. Combination targeting strategies could hence become a viable approach to maximize LNPs organ specificity and overcome limitations associated with each individual targeting approach in the future.

**FIGURE 8:**
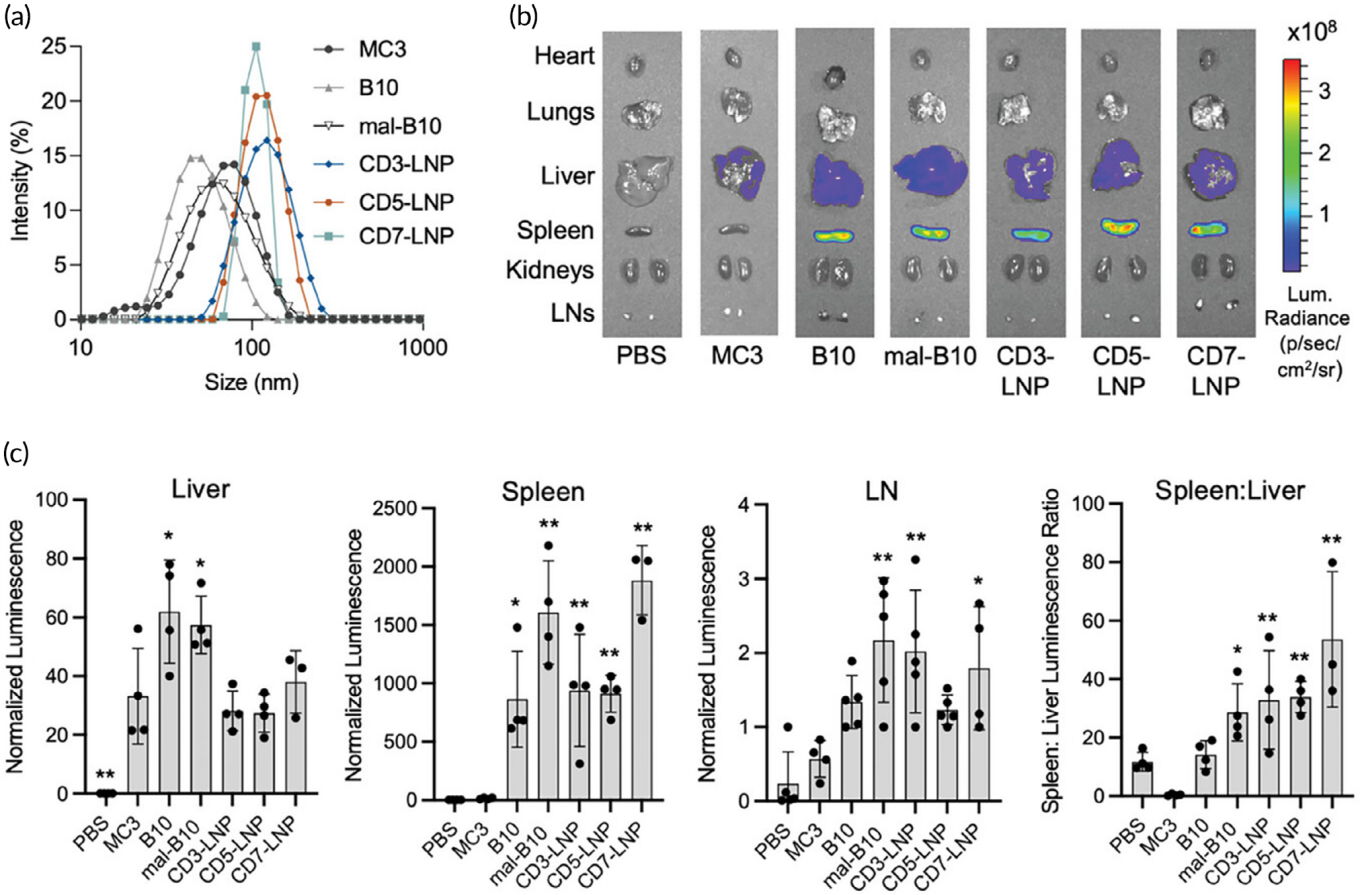
Antibody‐mediated lipid nanoparticles (LNP) targeting to the spleen. (a) Dynamic light scattering measurements of LNP size distribution with and without conjugation of T‐cell specific antibody fragments (anti‐CD3, anti‐CD5, anti‐CD7). (b) Representative IVIS images of organs harvested from mice 6 h after IV injection of conventional and antibody‐conjugated LNPs containing luciferase‐encoding mRNA. (c) Quantification of luminescence from ex vivo IVIS images of the liver, spleen, and lymph nodes (LNs) following LNP administration. *p<0.05, **p<0.01. This work was previously published by Wiley Publishing and reproduced under the terms of the CC BY‐NC‐ND 4.0 open access license.

Recently, there has been a rise in clinical stage in vivo CAR T‐cell therapies incorporating antibody fragment mediated active targeting of lentiviruses and LNPs (Table 1). Billingsley et al. achieved successful T‐cell targeting for in vivo CAR T‐cell generation using LNPs functionalized with reduced antibody fragments against CD3, CD5, and CD7, in which the Fc region was excluded^327^ (Figure 8). Fc exclusion comes with several benefits including reduced complexity, smaller size of the LNP‐antibody conjugate, and significantly reduced potential for Fc‐mediated opsonization and phagocytic clearance in case the antibody adopts a conformation with the Fc region facing outward from the surface of the LNP.^328^ The use of single chain variable fragments (scFv) is another viable alternative to full chain antibody grafts. scFv are fusion proteins in which only the antigen binding, variable regions of the heavy and light chains of an antibody are linked by a short peptide chain. scFv have demonstrated clinical safety and received FDA approval in the context of CAR T therapies.^329^, ^330^ Wong et al. reported that LNPs functionalized with scFv against prostate membrane antigen (PSMA) had a 1.6 fold improvement in tumor homing in a prostate cancer mouse model.^331^

Like their use for LNPs, antibodies have also been used for the targeting of PNPs to immune cells. While this has largely been used for PNP delivery in cancer, these same technologies can be applied to other applications. Specifically, there is the potential to use these targeted PNPs for the treatment of autoimmune conditions in which various immune markers are upregulated. Antibodies against these immune cell markers can be conjugated to PNPs containing nucleic acids to allow for specific delivery to these certain cell types. For example, Parayath et al. used in situ modification of PBAEs with CD64 targeting antibodies to treat lupus erythematosus.^332^ They found that using the targeting antibody resulted in a higher transfection of GILZ mRNA in myeloid regulatory cells in vitro.

### 4.2 Peptide‐mediated targeting

Functionalizing gene vectors with peptides is another viable option to circumvent the challenges posed by using full‐length antibodies. Like described in Section 3.1.4, screening of the most efficient peptide ligand to a given target is accessible through phage display, providing a fast, economical way of identifying viable targeting ligands. Yu et al. conducted a study with the aim of improving the ability of AAV2 to transduce striated muscle cells by incorporating a muscle‐targeting peptide into its capsid sequence.^333^ The change in capsid composition resulted in enhanced tropism to muscle cells in vivo, as well as a significant decrease in expression in non‐muscle tissues, indicative of successful re‐targeting of these vectors. Grifman et al. employed this strategy to improve the transduction and targeting capabilities of AAV2 toward tumor cells.^334^ Here, a tumor targeting sequence (NGR) was integrated into the capsid sequence and, as a result, these vectors displayed an increased tropism toward the cancer cells that overexpress the CD13 receptor.

Hagino et al. demonstrated successful lung targeting and improved endosomal escape using the membrane fusion peptide GALA (WEAALAEALAEALAEHLAEALAEALEALAA) to decorate pDNA‐loaded LNPs.^335^ This was demonstrated by improvement in lung/liver and lung/spleen uptake ratios. Similarly, Lou et al. screened various membrane active peptides to modify the surface of polyplexes.^336^ These polyplexes were used to deliver mRNA for vaccination. After screening, they identified GALA as the ideal peptide to conjugate to the polyplex, as it had the highest mRNA transfection in both macrophages and dendritic cells. This GALA‐modified polyplex showed strong promise as an mRNA vaccine platform, being able to stimulate and be taken up by various immune cells. In another study, Fornaguera et al. modified the ends of PBAEs with oligopeptides to deliver mRNA in polymer NPs for vaccination.^337^ The modified NPs showed successful targeting and efficient transfection in antigen‐presenting cells, which are key immune cells for vaccination.

Angiopep‐2 functionalization of NPs is useful for traversing the BBB as well as internalization by brain cancer cells. This is due to the binding that occurs between angiopep‐2 and lipoprotein receptor‐related protein 1.^338^ This protein is expressed in high quantities in both brain cancer cells such as glioblastoma and the endothelial cells of the BBB.^339^ Liu et al. capitalized on this affinity of angiopep‐2 to deliver two microRNAs for glioblastoma treatment.^340^ Angiopep‐2 decorated PNPs were able to effectively deliver these gene therapies, reducing tumor growth and increasing survival time. In a similar study, Shi et al. utilized an angiopep‐2 decorated polymersome delivery system to deliver siRNA across the BBB for the treatment of glioblastoma.^341^ These polymersomes showed enhanced circulation time and similarly reduced tumor growth and increased survival time in mice.

Arginylglycylaspartic acid (RGD) is an important peptide that has been widely studied in a variety of contexts from drug delivery in cancer to tissue engineering.^342^ RGD is an integrin‐binding domain, and this property has been exploited for targeted gene delivery. Similarly to angiopep‐2, RGD can be used to target glioblastoma and circumvent the BBB. Zhan et al. utilized an RGD modified PEG‐PEI to deliver a sensitizing gene and paclitaxel for glioblastoma treatment.^343^ Combining the paclitaxel and gene therapies resulted in decreased cell viability when compared to the paclitaxel or gene only nanocarriers. This trend was observed both in vitro and in vivo, suggesting that RGD modification of polymer nanocarriers as well as codelivery of chemotherapy and gene therapies is useful for glioblastoma treatment. Capitalizing on the binding of RGD to integrins, Kim et al. designed an RGD modified polymer nanocarrier to deliver gene therapy in tumor cells that overexpress integrin.^344^ This study showed that tumor growth was significantly inhibited in a mouse model and the carriers were taken up by micropinocytosis and clathrin‐mediated endocytosis. Exploring the endocytic pathways of various polymer NPs is critical to developing more effective gene therapies and this study shows the potential for treating various cancers that overexpress integrins.

Zheng et al. conjugated two peptides to the surface of a PNP to target siRNA to amyloid plaques for the treatment of Alzheimer's disease.^345^ When compared to NPs with none or only one of the two peptides, the NPs with both peptides were better able to penetrate the BBB and localize siRNA delivery to amyloid beta plaques. In another effort to treat Alzheimer's disease, Wang et al. conjugated a neural cell adhesion molecule‐mimetic peptide to a polymer NP encapsulating a gene therapy targeting TREM2 to re‐program dysfunctional microglia and slow the progression of disease.^346^ The results of this study were promising, as they increased amyloid beta plaque clearance, which can improve cognition. Recently, Cao et al. identified a peptide‐mediated strategy to generate BBB‐penetrating LNPs via targeting of the serotonin receptor, leading to a 50‐fold increase in mRNA delivery to the brain as compared to conventional LNPs.^347^ Similarly, Han et al. engineered peptide‐functionalized LNPs that target the acetylcholine receptor, leading to significant increases in mRNA delivery to the brain following systemic administration as well as improved transduction within neurons.^348^

Peptides have also been used to overcome the inherent cytotoxicity associated with PEI. Taheri et al. conjugated levo‐DOPA, an amino acid, onto high MW branched PEI which resulted in lower cytotoxicity than the unmodified PEI.^349^ L‐DOPA can also target L‐type amino acid transporter 1, which is useful in certain cancer types where this transporter is upregulated. This was demonstrated in murine mammary cancer cells, where 2.5‐fold higher transfection efficiency was observed for the L‐DOPA modified PEI NPs in comparison to the unmodified PEI NPs. Indolicidin has also shown promise as a peptide that can reduce cytotoxicity associated with PEI. Hu et al. created high MW PEI NPs with two different conjugation strategies for indolicidin, an antimicrobial peptide with immunomodulatory properties.^350^ Both conformations displayed higher transfection efficiency than unmodified PEI and lower cytotoxicity, although these NPs still showed cytotoxicity, especially at higher N/P ratios.

### 4.3 Aptamer‐mediated targeting

**FIGURE 9:**
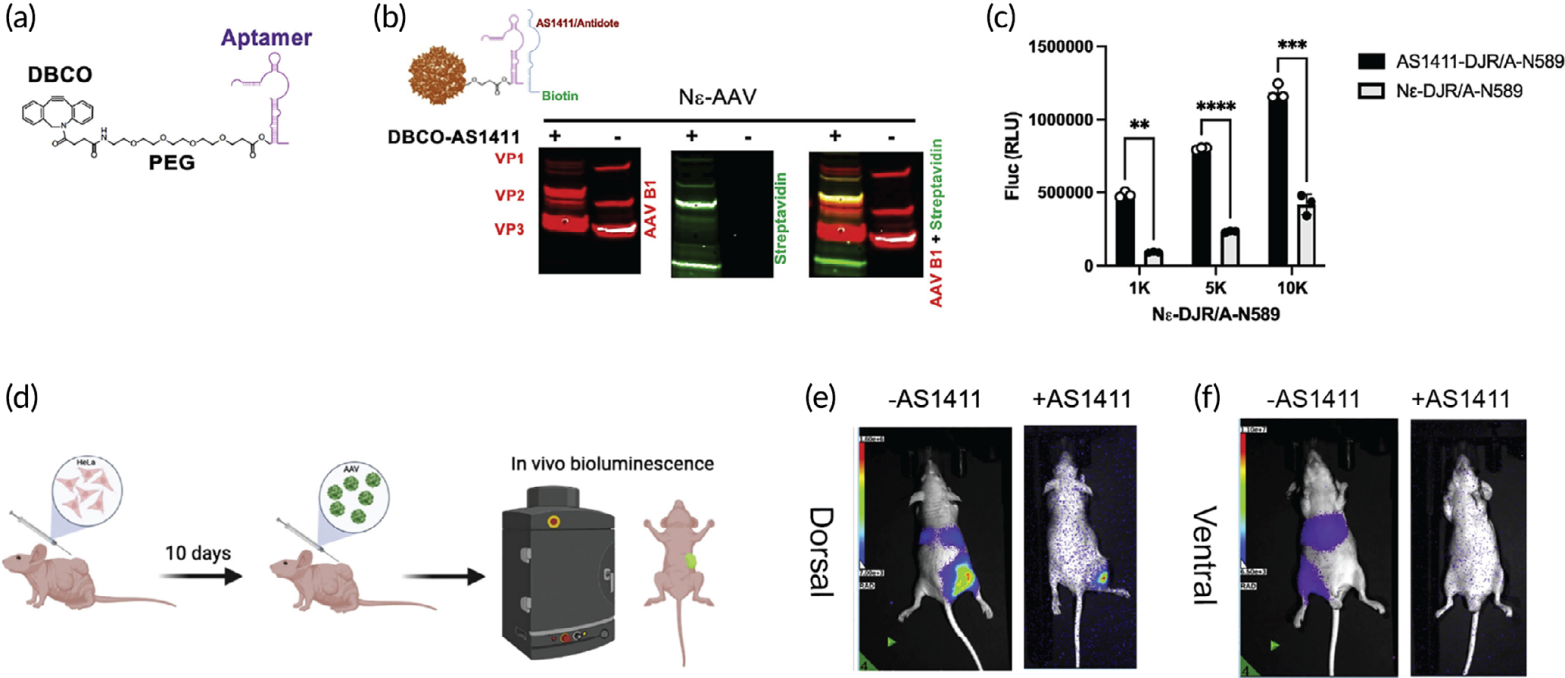
Aptamer‐mediated adeno‐associated virus (AAV) targeting to solid tumors. (a) Schematic representation of the dibenzocyclooctyne (DBCO)‐polyethylene glycol (PEG)‐aptamer (AS1411) used for receptor‐mediated cancer cell targeting. Azido‐lysine modified AAV vectors (Nε‐AAV) enable click chemistry‐mediated conjugation of DBCO‐PEG‐aptamer to the capsid surface. (b) Western blot analysis confirms successful conjugation of DBCO‐PEG‐AS1411 to Nε‐AAV capsid proteins (VP1, VP2, and VP3). (c) Luciferase activity in MCF‐7 breast cancer cells transduced with unconjugated and AS1411 conjugated Nε‐DJR/A‐N589 AAV vectors at multiplicity of infection (MOI) of 1000, 5000, and 10,000. *p<0.05, **p<0.01, ***p<0.001, ****p<0.0001. (d) Schematic representation of flank tumor in vivo model. (e) Dorsal and ventral (f) IVIS imaging of mice treated with 5 × 10^9^ vg of Nε‐AAV, FA‐AAV, and AS1411‐AAV 14 days after AAV treatment. Dorsal‐oriented imaging enables visualization of the tumor site on the hindlimbs of mice. Ventral‐oriented IVIS imaging enables visualization of off‐target liver transduction in the thoracic cavity. This work was previously published by the American Society of Gene & Cell Therapy and reproduced under the terms of the CC BY‐NC‐ND 4.0 open access license.

Aptamers are short synthetic ssDNA or ssRNA sequences that can bind varying biological moieties such as proteins, peptides, or other small molecules with high specificity. They can be a very useful tool for gene vector targeting due to their limited dimensions compared to polysaccharides, peptides, and proteins, which allows them to access sterically hindered receptors inaccessible to bulkier moieties. Approaches to aptamer‐mediated AAV gene therapies have been pursued in past studies. A study conducted by Puzzo et al. highlighted their approach to bind the tumor targeting DNA and RNA aptamers AS1411 and E3 RNA to the viral capsids of AAV^351^ (Figure 9). Upon incorporating that nucleotide sequence into the capsid of AAV, they then tested the effect of aptamer incorporation on transduction in vitro and in vivo. In their in vitro study, they observed a three‐ to nibe‐fold increase in transduction compared to AAV vectors that were devoid of the aptamers, and using specific competitors, they established that the transduction was indeed ligand‐specific. In vivo, they observed that there was selective uptake at the tumor site of these aptamer‐targeted AAV vectors without off‐target transduction within the liver. This study motivates further development of aptamer‐targeted AAV gene therapies which could increase the likelihood of effective treatment while minimizing off‐target effects.

Limited but promising data exist so far on the use of aptamers for LNP and PNP targeting. For example, Lee et al. developed DNA aptamer‐conjugated LNPs targeting PD‐L1 cancer cell receptors.^352^ The PD‐L1 DNA aptamer‐LNP could deliver phosphatase and tensin homolog (PTEN) mRNA to prostate cancer cells in vivo, leading to a 30% increase in apoptotic tumor cell populations in vivo compared to non‐functionalized LNPs. Tools like sequential evolution of ligands by exponential enrichment (SELEX) enable high‐throughput screening and selection of suitable aptamers for a specific target.^353^ Certain limitations such as the liability of single‐stranded NAs moieties serving as aptamers to degradation by extracellular nucleases, as well as their liability to trigger innate immune responses should be taken into consideration when designing aptamers. Aptamers convey a negative charge, and care should be taken that this does not interfere with endosomal escape abilities. Sanati et al. utilized aptamer targeting for the codelivery of chemotherapeutics and short hairpin RNA (shRNA) in PLA‐PEI micelles.^354^ They found that, in a murine tumor model, the aptamer‐conjugated micelles significantly inhibited tumor growth when intravenously administered. Aptamer targeting of LNP and PNP is perhaps not as advanced as other forms of active targeting, but has clearly shown potential to enhance organ‐targeted delivery.

### 4.4 Mono‐ and polysaccharide‐mediated targeting

Sugars are another class of molecules that have demonstrated preclinical as well as clinical success in LNP targeting. Except for Onpattro, all other FDA‐approved siRNA therapies utilize *N*‐acetyl‐D‐galactosamine (GalNAc) conjugated siRNA for hepatocyte targeting through the Asialoglycoprotein receptor (ASGPR).^355^, ^356^ However, as noted, all approved GalNAc siRNA bioconjugates for liver targeting are administered subcutaneously. As such, the passive, endogenous, active targeting mechanisms will differ substantially as compared to intravenously administered nanocarriers discussed throughout this review. Recently, Verve Therapeutics reported pausing three clinical trials utilizing passively targeted LNPs (Verve‐101) to deliver nucleic acids in atherosclerotic cardiovascular disease (ASCVD) and uncontrollably high low‐density lipoprotein cholesterol (LDL‐C) unresponsive to the current oral standard of care by permanently silencing PCSK9 gene. However, Verve Therapeutics has since initiated a second clinical trial in 2024, in which they replaced Verve‐101 with a GalNac decorated LNP (Verve‐102) with the same gene‐silencing cargo. GalNac functionalization is hypothesized to endow Verve‐102 access to hepatocytes through not only the LDL receptor, which may be downregulated in HeFH patients, but also through the ASPGR receptor. Importantly, this clinical trial could be the first to feature active targeted LNPs.

To generate PNP efficient at hepatic gene delivery, Wu and Wu investigated the transfection capabilities of a targeted PLL‐DNA complex on hepatocytes.^357^ To do this, they conjugated asialoorosomucoid (ASOR) to PLL, then complexed the altered PLL with DNA. ASOR is an asialoglycoprotein, a group of glycoproteins that specifically bind to hepatocytes. In this way, Wu et al. showed that gene delivery could be achieved in a specific manner. When tested in two cell lines, their PLL delivery system showed effective DNA transfection in HepG2 cells, but not SK‐Hep 1 cells. Mannose is another monosaccharide that has shown promise in targeting immune cells via their abundant CD206 receptor. Goswami et al. demonstrated that a self‐amplifying RNA vaccine encoding hemagglutinin delivered using mannosylated LNPs showed an accelerated adaptive immune response, higher antibody titer, and antigen specific CD8+ counts compared to non‐mannosylated LNPs following intramuscular administration.^358^ Mannosylated LNPs have also been shown to redirect LNPs from hepatocytes to LSECs (5× increase in transfection efficiency) following systemic administration.

Polysaccharides such as chitosan and hyaluronic acid have also been employed to direct nanocarriers to cells and tissues of interest. For example, Srivastav et al. utilized chitosan‐coated PLGA NPs to successfully deliver siRNA as well as CRISPR/Cas9, significantly increasing transfection when compared to non‐coated PLGA NPs.^359^ Using a different polymer as their base, Wang et al. conjugated transactivator of transcription (TAT)‐chitosan to the NP surface, improving the number of NPs that had successful delivery to the nucleus.^360^ These NPs contained both DNA and doxorubicin and induced apoptosis in a large percentage of cells. Zhao et al. coated chitosan‐PEI hybrid PNP with hyaluronic acid to investigate the change in transfection abilities of encapsulated siRNA.^361^ They found that the hyaluronic acid coated PNP resulted in a larger decrease in endometriotic lesions when compared to the non‐coated counterparts. In this case, the hyaluronic acid is also helpful as it binds to CD44, which is overexpressed in endometriotic cells.^362^ These studies suggest that hyaluronic acid is a useful coating modality for PNP. CD44, a receptor for hyaluronic acid, is often upregulated in tumors; therefore, coating with hyaluronic acid presents an inherent advantage for gene silencing and immunotherapy for applications in cancer. Guo et al. sought to improve the transfection efficiency of linear PBAE via a coating of hyaluronic acid.^363^ They found that the hyaluronic acid was able to increase the transfection efficiency of an encapsulated mRNA, improving by over 20%. These findings show the advantages of hyaluronic acid as a NP coating moiety.

### 4.5 Chemical modification‐mediated targeting

**FIGURE 10:**
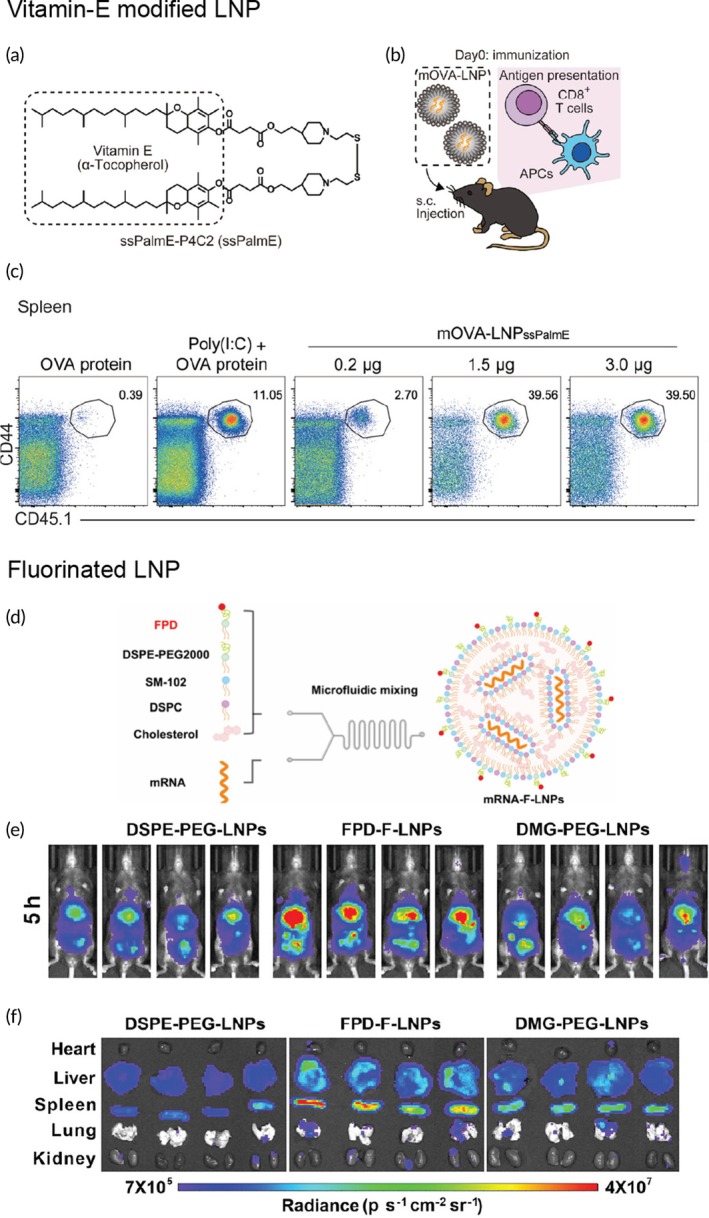
Vitamin E‐modified and fluorinated lipid nanoparticles (LNPs) for improved mRNA delivery. (a) Chemical structure of ionizable lipid with a vitamin E scaffold (ssPalmE‐P4C2; ssPalmE). (b) Schematic showing immunization of mice with ssPalmE‐containing LNP with mRNA encoding for ovalbumin (OVA). (c) Proliferation of CD8^+^ T‐cells in spleens of OT‐I T‐cell receptor transgenic mice 7 days after immunization with OVA, OVA + Poly(I:C), and OVA ssPalmE LNP analyzed using flow cytometry. (d) Schematic illustration of LNP formulations with the inclusion of fluorinated polyethylene glycol (PEG)‐DSPE (FPD). (e) IVIS imaging of live mice 5 h post‐systemic administration of LNPs containing DSPE‐PEG, DMG‐PEG, or FPD. (f) IVIS imaging of selected tissues 8 h post‐systemic administration of LNPs. The work shown in panels (a–c) was previously published by the American Chemical Society and reproduced under the terms of the CC BY‐NC‐ND 4.0 open access license. The work shown in panels (d–f) was reproduced with permission from the American Chemical Society.

Scaffolding NP components with vitamin E has been shown to augment their uptake beyond endocytosis by utilizing additional receptors such as ABCA1, SR‐BI, and NPC1L1. Additionally, vitamin E has been reported to possess antiangiogenic properties,^364^ limiting tumor proliferation as well as immunomodulatory properties.^365^ Hence, the inclusion of vitamin E in the NP structure can endow some of its functional roles of LNPs and PNPs. Vitamin E scaffolded or derived ionizable lipids have been shown to augment the cytotoxic T‐cell activation upon their incorporation into LNP‐delivered mRNA vaccines. Oyama et al. demonstrated that using alpha‐tocopherol scaffolded ionizable lipid (ssPalmE) in combination with DOPE, cholesterol, and DMG‐PEG2000 gave rise to LNPs with improved capacity for cellular immunity induction in a murine OVA tumor model, as well as a Toxoplasmosis gondii infection model^366^ (Figure 10a–c). They could elucidate that alpha‐tocopherol promoted CD8+ T‐cell activation by enhancing type I interferon‐dependent innate immune responses, as well as selectively transfecting conventional dendritic cells‐2 (cDC2) involved in skin‐to‐lymph node migration and subsequent cross‐presentation. Similar results were demonstrated using ssPalmE by Anindita et al.^367^ Cui et al. further explored this concept iteratively, by designing an ionizable lipid's library with vitamin E derivatives as hydrophobic tails.^368^ They elucidated the structure activity relationship of the ionizable lipids with the resulting LNPs' immunomodulatory properties. Through their study they demonstrated that vitamin E containing LNPs had enhanced uptake compared to conventional LNPs through VE‐associated receptor proteins in lipid rafts or caveolin and could affect DC maturation through activating the STING pathway. They showed that reducing the distance between the two N atoms of the head group as well as the steric hindrance in the tail structure, as well as the introduction of hydroxyl head groups were conductive of higher transfection.

Vitamin E has been used to coat PNP for gene delivery, specifically PEI polyplexes. Liu et al. showed that vitamin E labeled PEI was able to complex with pDNA and showed higher transfection efficiency in three cell lines in vitro and in mice in vivo at a variety of PEI to DNA ratios.^369^ This study used a branched 25 kDa PEI as the control without vitamin E modification, which was found to be less effective than the vitamin E conjugated 1.8 kDa branched PEI. In another study using the same PEI types, Ren et al. developed an mRNA vaccine as an alternative to more common lipid NP platforms in an effort to overcome unwanted side effects.^370^ At higher N/P ratios, wherein there is a higher ratio of PEI to mRNA, the vitamin E modified PEI polyplexes showed significantly higher transfection ability across three cell lines.

Caprolactone (or ε‐caprolactone) is a chemical that has shown promise in polymeric drug delivery when conjugated to other polymers.^371^ Caprolactone has also been used to improve polymeric delivery of gene therapies. PBAE and PEI, two of the most common polymers used in delivering gene therapies, have both been modified with caprolactone with promising results. Capasso Palmiero et al. found that varying the number of caprolactone units appended to a PBAE terpolymer greatly alters the physical and chemical properties of the polymer.^371^ Utilizing a PBAE library for screening, they found that, similarly to the conclusions above, the formulation used for PBAE complexation with oligonucleotides greatly impacts nucleic acid delivery abilities. Using the caprolactone‐modified PBAE, the authors were able to achieve a much higher transfection ability of mRNA than in PEI. Caprolactone was also used to modify PEI for the delivery of siRNA. When compared to an unmodified PEI, the caprolactone‐modified PEI was able to efficiently silence genes in addition to simultaneously loading siRNA and DNA for in vitro tests, which could prove useful for developing more complex gene therapies.^372^

Lv et al. attached fluorinated ligands onto polymers in an attempt to increase transfection efficiency of encapsulated gene therapies.^373^ This modification of polymer NPs resulted in an increase in cellular uptake, ultimately leading to an increase in transfection efficiency. Fluorination helped NPs to cross the lipid bilayer into the cell, as the ligands were both hydrophobic and lipophobic, characteristics that enable membrane crossing. Fluorination can also be used to reduce cytotoxicity of PEI, one of the main drawbacks to this widely used gene delivery system. Xue et al. found that fluorination of high MW (25 kDa) branched PEI significantly reduced cytotoxicity.^374^ In addition, when the PEI NPs containing siRNA were administered in mice, fluorination of the PEI altered the site of accumulation of the NPs. For the non‐fluorinated particles, accumulation was most widely seen in the lungs, while the fluorinated NPs mainly accumulated in the liver. These findings suggest that fluorination may be used for extrahepatic organ targeting of PNPs.

In addition to increased biocompatibility, fluorination has also been found to increase transfection efficiency in various cell lines. Gong et al. fluorinated PBAEs and compared them to non‐fluorinated PBAEs, 25 kDa PEI, as well as Lipofectamine, the commercial standards for transfection.^375^ The transfection efficiency of the fluorinated PBAE outperformed the other methods of gene delivery. A similar trend has been observed for LNPs, where fluorination of lipid components of an LNP can endow the LNP surface with higher stability and lipophilicity contributing to enhanced uptake and endosomal escape due to the robustness of the fluorine carbon bond. Zhang et al. showed that fluoridation of DSPE‐PEG‐2000 incorporated into LNPs at its usual molar contribution of only 1.5% could affect a two and five‐fold increase in level of reporter protein expression in B16F10 tumor cells as well as dendritic cells in vitro, while promoting up to a three‐fold increase in level of reporter protein expression following IV or IP administration in comparison to non‐fluorinated LNP controls, with specific enhancement splenic expression (Figure 10d–f).^376^ Recently, Chen et al. took this one step further by developing ultrasound‐assisted fluorinated PEGylated LNPs.^377^ Where these particles could effectively target the spleen and then using ultrasound, timely shedding of PEG could be induced. This strategy showed a four‐fold increase in splenic transfection and a more potent cellular immune response in ovalbumin‐expressing B16F10 tumors in vivo. Huo et al. also demonstrated enhanced splenic delivery using fluorinated LNPs, only this time the fluorinated component was the ionizable lipid. By combining fluorinated and hydrocarbon counterparts of the same ionizable lipid in an appropriate ratio enhanced the encapsulation and transfection efficiencies of LNPs both in vitro and in vivo. They also demonstrated splenic targeting following IV injection of the fluorinated LNPs.

## 5 CONCLUSIONS AND FUTURE DIRECTIONS

The continued growth of gene therapies as treatments for a broad range of diseases hinges on overcoming the challenge of precise and effective delivery. While AAV and LNP represent the most clinically advanced platforms for gene delivery, their tendency to accumulate in the liver and potential for undesired immunological response highlights the need for alternative formulation strategies. Broadening the reach of gene therapies to extrahepatic targets will require a diverse and adaptable repertoire of delivery systems that build on recent advances in adeno‐associated viral and NP engineering. While passive and active targeting have their advantages, protein corona‐mediated endogenous targeting strategies represents an emerging and highly valuable approach. Harnessing endogenous targeting for organ‐targeted gene therapy will require further advances in biophysics, colloidal science, chemistry, and pharmaceutical engineering to fully recognizes its potential to fine‐tune gene vector biodistribution in target tissues.^378^, ^379^ While most extensively studied and leveraged for LNP based gene delivery, this concept is now being applied to PNPs as well as AAVs, with the potential to transform targeting strategies across these platforms. Beyond enhancing payload delivery to target sites, endogenous targeting may help to reduce off‐target effects and improve the safety profile of future gene therapies. Future work must also address how endogenous targeting operates for other modes of administration (e.g., oral, inhaled, and subcutaneous) and how patient‐specific variations, such as disease‐driven changes in protein corona composition, affect outcomes.^380^, ^381^, ^382^ Looking ahead, delivery systems may advance beyond providing spatial control of gene delivery in target organs by incorporating temporal control, enabling sustained, pulsatile, or staged release reminiscent of controlled release drug delivery systems.^383^, ^384^, ^385^, ^386^, ^387^ Another cornerstone of next‐generation nanocarrier design is high‐throughput screening. By rapidly testing large design spaces, these methods reduce the time and resources needed to identify promising candidates. The integration of artificial intelligence can further accelerate this process,^388^, ^389^, ^390^ though current computational models are limited by the bias of published data sets, which often underreport negative results. Capturing both successes and failures will be essential for building robust predictive computational frameworks. Taken together, progress in targeting strategies, automated screening, and computational modeling underscores how far platforms for gene therapy have advanced in the past decade—and how much potential remains for expanding their reach and refining their performance.

## AUTHOR CONTRIBUTIONS

GAD, SSN, YC, AS, OT, and SP worked collaboratively to conceptualize the review article scope and content to be included. All authors contributed to the writing and editing of the manuscript.

## CONFLICT OF INTEREST STATEMENT

The authors declare no competing interests.

## Data Availability

This article is a review of previously published literature. No new data were generated or analyzed; therefore, data sharing is not applicable.
